# In vitro and in silico studies and a systematic literature review of antiglycation properties of amlodipine

**DOI:** 10.1038/s41598-025-18925-8

**Published:** 2025-09-26

**Authors:** Karolina Dańkowska, Miłosz Nesterowicz, Kamil Klaudiusz Lauko, Daria Trocka, Małgorzata Żendzian-Piotrowska, Jerzy Robert Ładny, Anna Zalewska, Marta Żebrowska-Gamdzyk, Mateusz Maciejczyk

**Affiliations:** 1 Students’ Scientific Club “Biochemistry of Civilization Diseases Department of Hygiene, Epidemiology and Ergonomics , Medical University of Bialystok , 2c Mickiewicza Street, 15-233 Białystok, Poland; 2 Department of Hygiene, Epidemiology and Ergonomics , Medical University of Bialystok , Białystok, 2c Mickiewicza Street, 15-233 Poland; 3 Department of Emergeny Medicine , Medical University of Bialystok , Białystok, 24a M. Sklodowskiej-Curie Street, 15-274 Poland; 4 Independent Laboratory of Experimental Dentistry , Medical University of Bialystok , Białystok, 24a M. Sklodowskiej-Curie Street, 15-274 Poland; 5 Department of Dietetics Faculty of Health Sciences, Łomża Academy, Łomża, 14 Akademicka Street, 18-400 Poland

**Keywords:** Cardiac device therapy, Drug development, Blood proteins

## Abstract

Protein glycation is crucial in the pathogenesis of diabetes and its cardiovascular complications. Little is known about the antiglycation properties of amlodipine, a long-acting calcium channel blocker used to treat high blood pressure. In our study, amlodipine’s antiglycoxidant activity was assayed in sugars (glucose, fructose, and ribose), aldehydes (glyoxal), and chloramine T-modified bovine serum albumin (BSA). Aminoguanidine and N-acetylcysteine were used as standard glycation/oxidation inhibitors. The content of oxidation, glycoxidation, and glycation protein products was measured colorimetrically and fluorimetrically. A one-way analysis of variance (ANOVA) followed by Tukey’s post hoc test was used for statistical analysis. The mechanism of amlodipine’s antiglycoxidant activity was also evaluated using in-silico molecular docking. Amlodipine protects against BSA oxidation, as evidenced by enhanced total thiol content and mitigated protein carbonyls/advanced oxidation protein products. Amlodipine also increased the fluorescence of tryptophan and decreased the contents of kynurenine, N-formylkynurenine, and dityrosine. In addition, amlodipine effectively prevents protein glycation, as evidenced by a reduction in amyloid-beta structure, Amadori products, and advanced glycation end products (AGEs). In in silico analysis, amlodipine’s antiglycation properties were indicated during its interaction with BSA, glycosidases, and AGEs/receptor for AGEs (RAGE) pathway proteins. Among all proteins, amlodipine docked best with c-Jun N-terminal kinases. Summarizing, we have demonstrated the anti-glycation and antioxidant activity of amlodipine *in vitro.* This effect may be particularly important in patients with diabetes and atherosclerosis, where excessive glycation accelerates the development of vascular complications. Further studies are needed to confirm the antidiabetic activity of amlodipine in vivo.

**Supplementary Information:**

The online version contains supplementary material available at 10.1038/s41598-025-18925-8.

## Introduction

Hypertension remains the leading cause of premature death worldwide. In 2023, 1 in 5 women and 1 in 4 men had hypertension, which gives about 1.28 billion people^1^. This is of particular concern because hypertension is a significant risk factor for heart, brain, kidney, and other diseases^2,3^. Hypertension is also closely associated with diabetes^4^. Both hypertension and diabetes lead to endothelial dysfunction characterized by increased oxidative stress and activation of receptors for advanced glycation end products ([AGEs] RAGE). AGEs/RAGE stimulation activates immune cells, resulting in NADPH oxidase (NOX) overexpression, which is a primary cytoplasmic source of free radicals and pro-inflammatory factors such as tumor necrosis factor-α (TNF-α), interleukin 1β (IL-1β) and interleukin 6 (IL-6)^5–8^. Several studies observed that soluble RAGE (sRAGE) and full-length RAGE (flRAGE) expression was negatively associated with plasma glucose concentrations^9^. A decrease in flRAGE levels was observed in patients with impaired glucose tolerance and also diabetes, which was probably due to hyperglycemia-induced augmented proteolytic degradation of the RAGE subdomain mediated by disintegrins and metalloproteinases^10^. The formation of hyperglycemia-induced calcification under the impact of the AGEs/RAGE axis was also noted^11^. AGEs may cause vascular dysfunction by making cross-bonds between extracellular matrix proteins (elastin and collagen), which leads to blood vessel stiffness, heart fibrosis, and diastolic dysfunction^12^. Other proteins, such as low-density lipoproteins (LDL), are also susceptible to cross-linking with AGEs. Briefly, macrophages derived from monocytes take up glycated LDL, thereby stimulating the formation of foam cells that cause atherosclerosis. AGEs may also promote vascular dysfunction by reducing the bioavailability of nitric oxide (NO) and inhibiting endothelial NO synthase and antioxidant defense mechanisms^13,14^. Since inflammation and protein glycation play a vital role in the development of micro- and macrovascular complications, the therapy of hypertensive/diabetic patients should be focused on the modification of cardiovascular risk factors^4^.

**Fig. 1:**
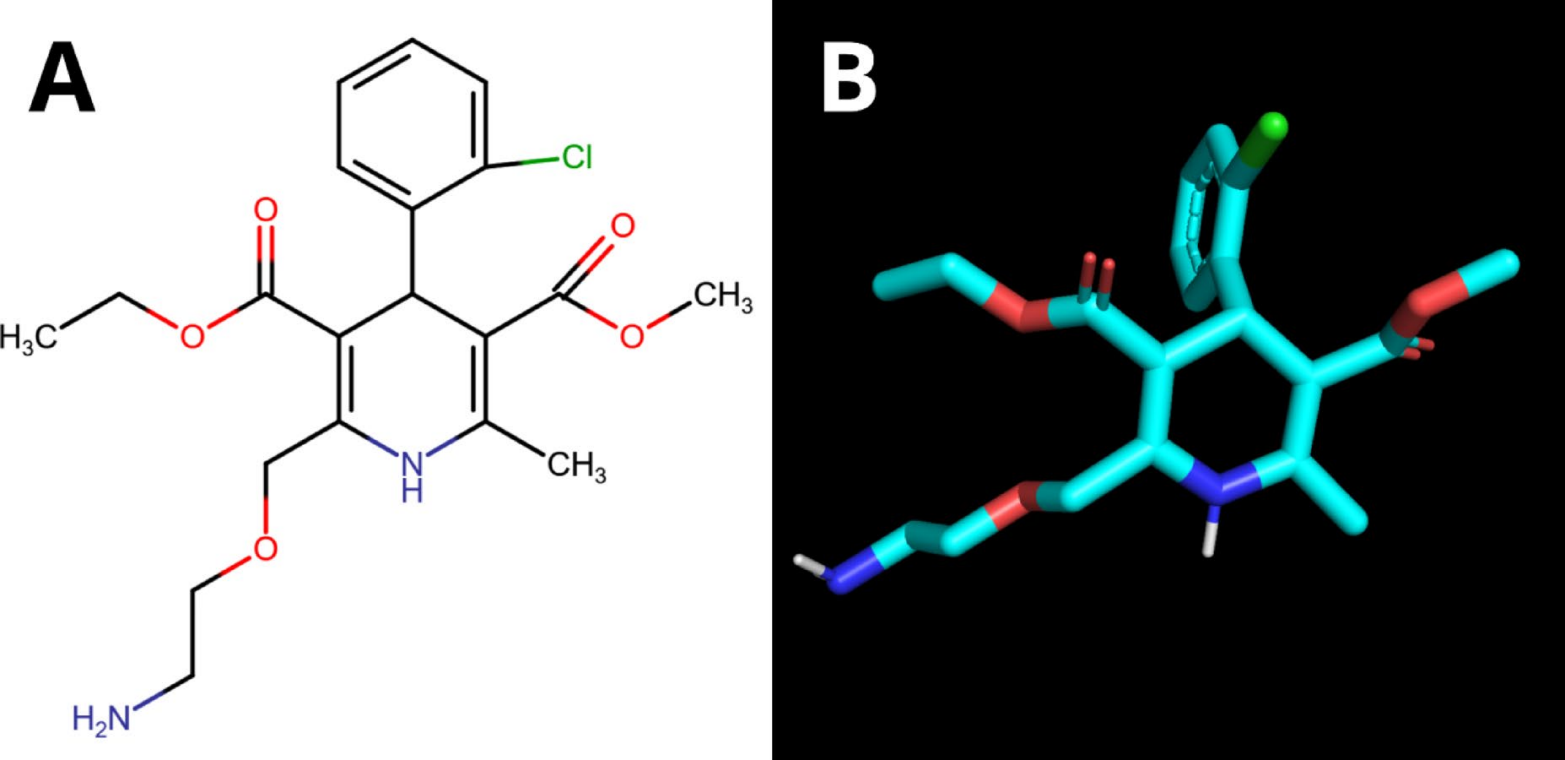
The structural formula (**A**) and the spatial structure (**B**) of amlodipine (3-*O*-ethyl5-*O*-methyl2-(2-aminoethoxymethyl)−4-(2-chlorophenyl)−6-methyl-1,4-dihydropyridine-3,5-dicarboxylate).

One of the most commonly used drugs for hypertension is amlodipine^15^. Amlodipine (3-*O*-ethyl5-*O*-methyl2-(2-aminoethoxymethyl)−4-(2-chlorophenyl)−6-methyl-1,4-dihydropyridine-3,5-dicarboxylate; C_20_H_25_ClN_2_O_5;_ Fig. 1) is a calcium channel blocker, a dihydropyridine (DHP) derivative from a nifedipine group^16,17^. The drug blocks the trans-membrane influx of calcium ions by inhibiting free voltage-dependent L-type calcium channels in vascular smooth muscles. This decreases intracellular calcium concentration, which reduces vascular smooth muscle tone, namely arterial vasodilation. Indications for amlodipine include left ventricular hypertrophy, asymptomatic atherosclerosis, angina pectoris, vasospastic angina, peripheral artery disease, metabolic syndrome, and, in the elderly, isolated systolic hypertension^18–20^.

Amlodipine is a highly effective drug used in the treatment of hypertension^21–23^. However, available literature suggests that amlodipine may have additional mechanisms of action, including reduction of oxidative stress and protein glycation, which play essential roles in the pathogenesis of cardiovascular diseases^21–23^. Compared to baseline and single monotherapies, a decrease in lipoprotein (a) and isoprostanes was found during the fixed combination of olmesartan and amlodipine^24^. However, the drug’s antiglycation properties are not well known. Our study is the first to comprehensively assess the antiglycoxidant effect of amlodipine, which could allow its use not only in hypertension. Carbonyl stress and endothelial dysfunction entail further pathologies leading to metabolic syndrome^25,26^. The antiglycoxidant effect of amlodipine, on the one hand, may have antidiabetic potential and, at the same time, protect against cardiovascular diseases. Thus, the multifaceted nature of this drug may allow for a holistic approach to treatment.

## Materials and methods

### Systematic review

The Literature review covered January 2002 to May 2023 in the medical database (PubMed). The search terms we used to find these articles were: [amlodipine and oxidative stress], [amlodipine and antioxidative properties], [amlodipine and carbonyl stress], [amlodipine and ROS scavenging], [amlodipine and nitrosative stress], [amlodipine and antiglycoxidative properties], [amlodipine and antiglycation properties] and [amlodipine and protein glycation]. The inclusion criteria were as follows: publications in English, results collected from human studies and in vivo, in vitro experimental studies, systematic review articles, and publications reporting the antiglycoxidant effects of amlodipine. The exclusion criteria were publications in another language, publications not describing the antiglycoxidant effect of amlodipine, and case reports.

The authors conducted data pre-selection independently by assessing the titles and abstracts of the manuscripts (K.D., M.N.). Subsequently, a comprehensive review of the selected articles’ content was performed, and those meeting the inclusion and exclusion criteria were incorporated into the article. Any uncertainties regarding the content value of the articles were resolved through consultation with other authors (K.K.L.). The reliability level of the researchers was assessed using Cohen’s kappa coefficient (κ), which yielded a value of κ = 0.9. To ensure data quality, all publications underwent evaluation for methodology, with the following variables being distinguished: authors, study design, study duration, year of publication, study population size, inclusion and exclusion criteria, and research findings.

### Reagents and equipment

All chemical reagents used in the experiment were of analytical grade. BSA was procured from Fisher BioReagents (Pittsburgh, PA, USA), and the rest of the reagents were procured from Sigma-Aldrich (Numbrecht, Germany/Saint Louis, MO, USA). Before use, all solutions were sterilized by filtration through 0.2-µm membrane filters. Absorbance and fluorescence measurements were obtained using an Infinite M200 PRO multimode microplate reader (Tecan Group Ltd., Männedorf, Switzerland).

### Bovine serum albumin (BSA) model

Bovine serum albumin (BSA) glycation and oxidation were conducted using a previously established methodology^27–34^. Initially, with a purity of 98%, BSA was dissolved in a sodium phosphate buffer (0.1 M, pH 7.4) containing 0.02% sodium azide as a preservative. To induce glycation, sugars including glucose (Glc), fructose (Fru), and ribose (Rib) were employed, in addition to aldehydes such as glyoxal (GO). BSA was incubated with 1 mM amlodipine and 0.5 M Glc, Fru, and Rib for six days or with 2.5 mM GO for 12 h to measure their influence. GO was used within One month of delivery, and working solutions were prepared briefly before assessment. 20 mM chloramine T (ChT) was introduced as an oxidizing factor and incubated with BSA and amlodipine for one hour. Incubation was conducted in hermetically sealed vials in the dark with continuous vibration (50 rpm, 37 °C) for six days with sugars, 12 h with GO, and 1 h with ChT. The incubation mixtures included BSA at a final level of 0.09 mM^27–34^.The concentrations of glycation agents and the optimal incubation conditions for evaluating the modification of the glycoxidation rate by additives were established and validated based on prior kinetic studies^27,28^. Although the concentrations of sugars, aldehydes, and oxidants in the experiments exceeded physiological levels, they were instrumental in simulating physiological processes occurring over weeks or even months in the human body within a relatively condensed timeframe. These experimental conditions are routinely employed to appraise the antiglycation properties of newly investigated substances^27–34^. For comparative purposes, aminoguanidine, a recognized protein oxidation inhibitor, and N-acetylcysteine (NAC), an antioxidant, were employed alongside amlodipine. The concentration of all additives was standardized at 1 mM, aligning with the precedent established by other in vitro studies and proportion to the high concentrations of glycation agents^27–34^. The study was executed in three series, and each series was duplicated.

**Fig. 2:**
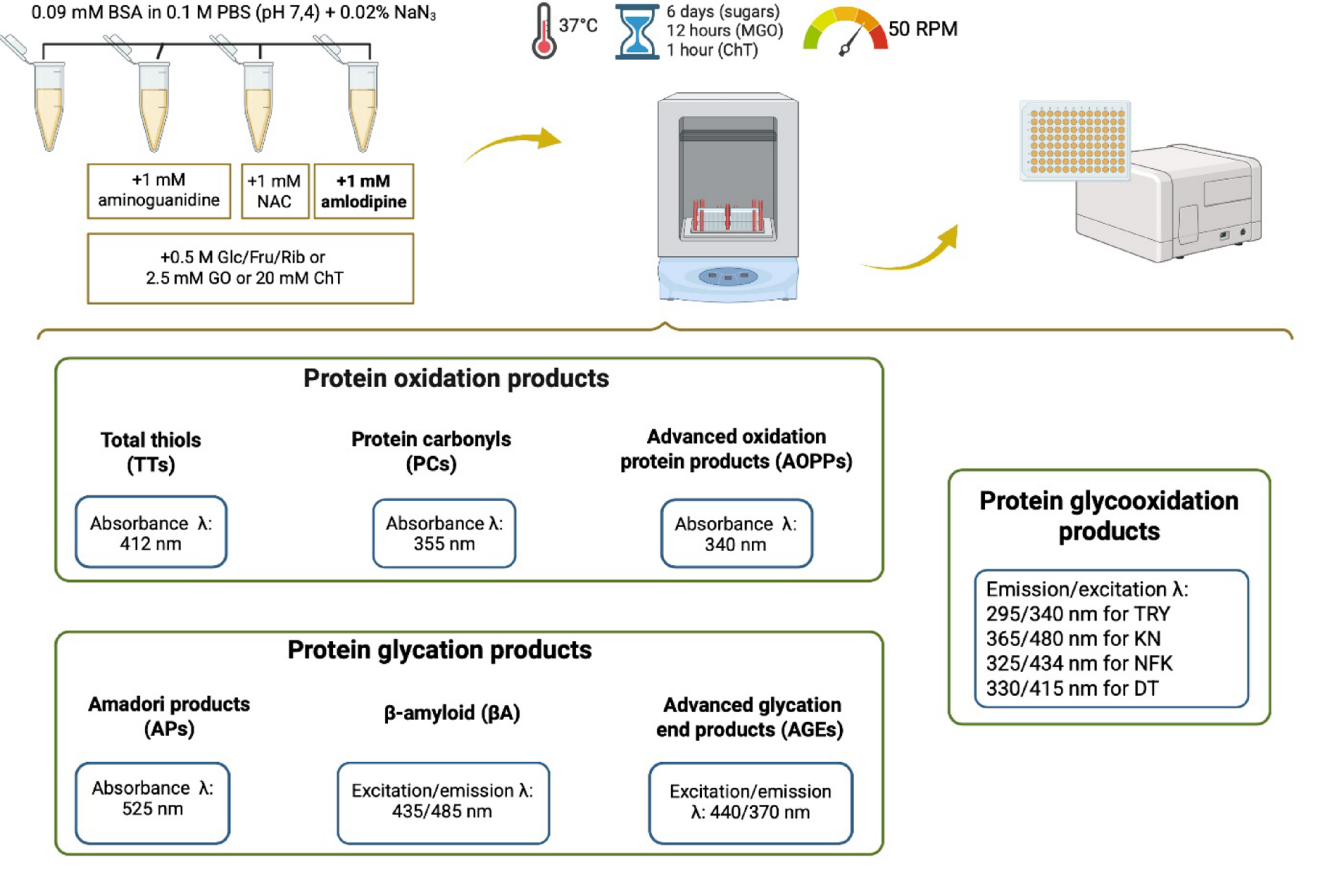
Graphical diagram of the methodology. Created with *BioRender.com*. BSA - bovine serum albumin; ChT - chloramine T; DT - dityrosine; Fru - fructose, Glc - glucose; GO - glyoxal; KN - kynurenine, NAC - N-acetylcysteine; NFK - N-formylkynurenine; PBS - phosphate buffered saline, Rib - ribose; TRY - tryptophan.

The methodology used in the study is presented in Fig. 2.

### Protein oxidation products

#### Total thiols (TTs)

Total thiols were assessed using a colorimetric method at a wavelength of 412 nm with the application of Ellman’s reagent. The concentration of thiol groups was calculated using a standard curve derived from reduced glutathione (GSH)^35^.

#### Protein carbonyls (PCs)

The protein carbonyl (PC) concentration was quantified through the reaction between carbonyl groups and 2,4-dinitrophenylhydrazine (DNPH) within oxidatively damaged proteins. The resulting reaction product’s absorbance was assessed colorimetrically at a wavelength of 355 nm. For quantification purposes, the absorption coefficient for 2,4-DNPH (22,000 M^−1^cm^−1^) was employed^36^.

### Advanced oxidation protein products (AOPPs)

Advanced oxidation protein products (AOPPs) levels were assessed through a spectrophotometric assay. To execute this, 200 µL of the examined samples, previously diluted with PBS at a 1:5 ratio (v/v), were combined with standard solutions ranging from 0 to 100 µmol/L alongside 200 µL of blank PBS solution. Then, 10 µL of 1.16 M potassium iodide and 20 µL of acetic acid were introduced into each well. The absorbance was promptly recorded at a wavelength of 340 nm utilizing a microplate reader and compared against the absorbance of the blank solution^37^.

### Protein glycoxidation products

The contents of tryptophan (TRY), kynurenine (KN), N-formyl-kynurenine (NFK), and dityrosine (DT) were assessed through fluorescence measurements utilizing specific emission and excitation wavelengths. The fluorescence emissions were recorded at 295 nm for TRY, 365 nm for KN, 325 nm for NFK, and 330 nm for DT. Correspondingly, the excitation wavelengths used were 340 nm for TRY, 480 nm for KN, 434 nm for NFK, and 415 nm for DT. Before the measurements, the solutions under investigation were appropriately diluted with 0.1 M H_2_SO_4_ at 1:5 (v/v). The outcomes were normalized employing the fluorescence intensity of 0.1 mg/mL quinine sulfate in 0.1 M H_2_SO_4_ as a reference^38^.

### Protein glycation products

#### Amadori products (APs)

Amadori product levels (APs) were assessed using a colorimetric Nitro Blue Tetrazolium (NBT) assay. The absorbance was gauged at a wavelength of 525 nm, and the calculation was grounded On the monoformazan extinction coefficient of 12,640 M^−1^cm^−1^^39^.

#### β-amyloid (βA)

Thioflavin T assessment was performed to detect the fluorescence emitted upon the interaction of amyloid fibrils or oligomers with thioflavin T. In this procedure, 90 µL of the samples were blended with 10 µL of thioflavin T and deposited into a microplate. The fluorescence intensity was gauged at a wavelength of 435 nm for excitation and 485 nm for emission^40,41^.

### Advanced glycation end products (AGEs)

The content of AGEs was determined through spectrofluorometry. The fluorescence specific to AGEs was recorded at an excitation wavelength of 440 nm and an emission wavelength of 370 nm using a 96-well microplate reader. Before measurement, the samples were appropriately diluted with PBS at a ratio of 1:5 (v/v)^42,43^.

### Molecular docking analysis

Molecular docking is a computational technique in silico to predict the preferred binding position of a ligand, like amlodipine, to a macromolecule, such as bovine serum albumin (BSA)^44,45^. This study employed BSA as the receptor in the interaction analysis with the amlodipine molecule. This inquiry into the potential binding of the drug to BSA provides valuable insights into its mechanism of action in safeguarding against carbonyl stress. The three-dimensional structure of BSA (PDB ID: 4F5S) was obtained from the Protein Data Bank (PDB) website (https://www.rcsb.org/) in.pdb format. This crystal structure was determined through X-ray diffraction at a resolution of 2.47 Å^31,32^.

Additionally, the three-dimensional structure of amlodipine (PubChem CID: 2130) was sourced from the National Library of Medicine website (https://pubchem.ncbi.nlm.nih.gov/) in.sdf file format. The BSA molecule underwent preparation using AutoDock MGL Tools. This involved the removal of all water molecules and the addition of polar hydrogens, as well as Kollman charges, to minimize energy. The processed protein structure was then saved in.pdbqt format. Subsequent molecular docking simulations were carried out employing AutoDock Vina, with a grid size of 40 × 40 × 40 and a spacing of 0.375 Å. The grid box was centered at the coordinates (x, y, z): 34.885, 23.976, and 98.792, respectively. An exhaustiveness parameter value of 8 was selected for the docking process. Finally, the resulting molecular docking interactions were visualized using PyMOL 2.5^31^^,^^32^.

### Statistical analysis

The results were expressed as a percentage relative to the corresponding control values (BSA + glycation agents: Glc, Fru, Rib, GO; or oxidizing agent: ChT). The equation used to calculate percent of control was following: % of control = value in sample × 100%/value in control. Group comparisons were evaluated using one-way analysis of variance (ANOVA), followed by Tukey’s post hoc test for multiple comparisons. A meaning level of *p* < 0.05 was deemed statistically considerable. Additionally, adjusted p-values were computed to accommodate multiple comparisons. Statistical Analysis was performed using GraphPad Prism 9 software (GraphPad Software, La Jolla, CA, USA).

## Results

### Systematic review

**Fig. 3:**
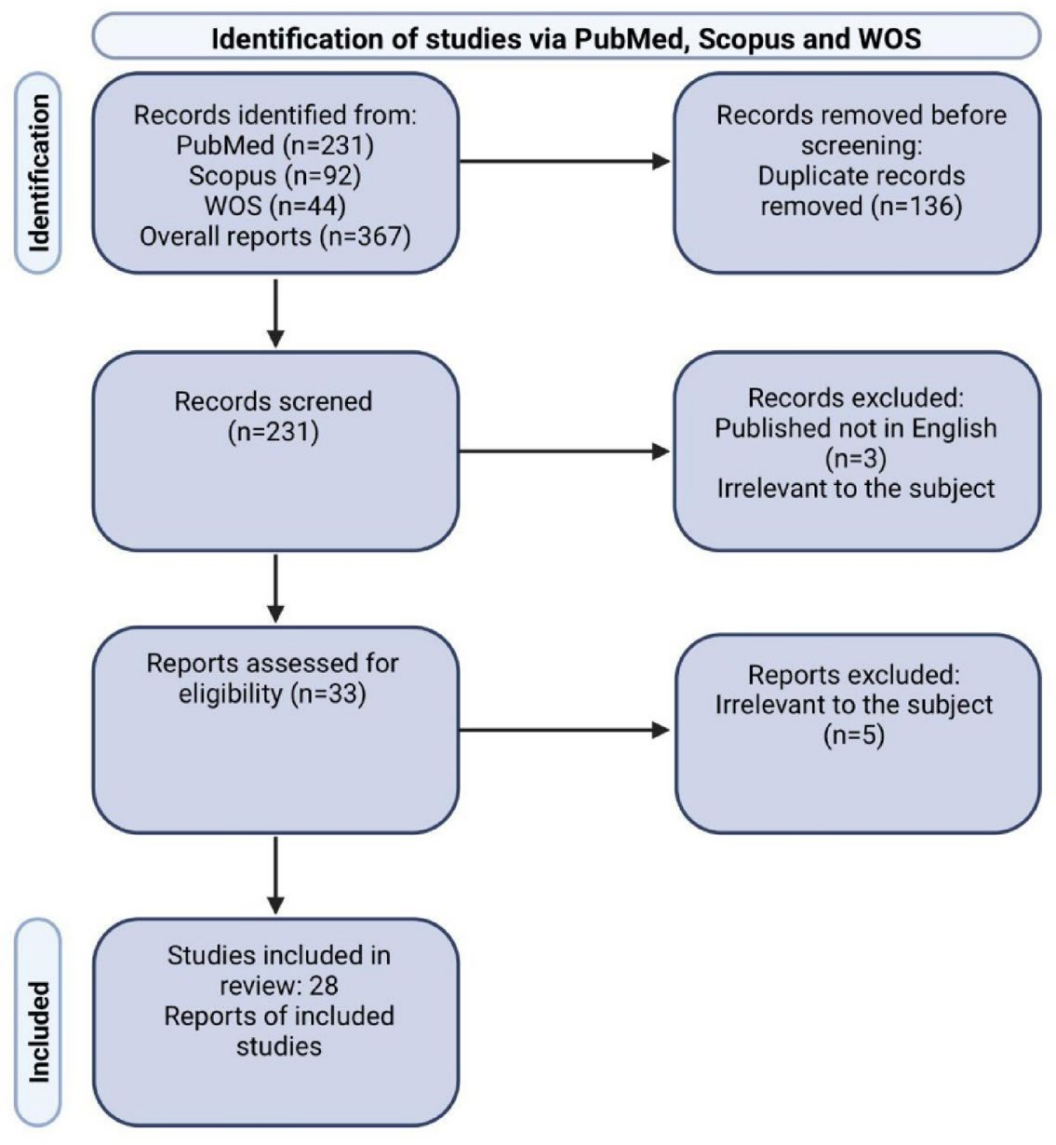
Flow diagram: Systematic review methodology (Prisma). WoS, Web of Science.

The Literature review revealed 34,745 works from the PubMed, Medline, and Web of Science databases, of which 32,462 were removed due to duplication. Two thousand two hundred eighty-three abstracts were read, and 218 met the inclusion and exclusion criteria. Among the qualified articles, 154 turned out to be irrelevant to the subject of our review. Therefore, 65 papers were finally included. The workflow diagram presenting the search strategy is shown in Fig. 3.

The vast majority of studies included in the review focused on comparing DHP calcium channel blockers with hypotensive drugs from other groups (e.g., angiotensin-converting-enzyme inhibitors [ACE-Is], angiotensin receptor blockers [ARBs], mineralocorticoid receptor antagonist [MRAs], thiazide diuretics), various substances in the DHP calcium channel blocker group, or using them in combination. Only three studies focused on exclusively testing amlodipine using various biomarkers of oxidative stress. The methodology and endpoints of all studies reviewed are presented in Table S1.

The positive effects of amlodipine on various biomarkers of oxidative stress have been widely documented. These include its antioxidant effects, protection against albumin glycoxidation, and protein glycation.

Three hundred sixty-seven papers were identified during the Literature review from the PubMed, Web of Science, and Medline databases, of which 136 were eliminated due to duplication. 231 abstracts met the inclusion and exclusion criteria. Of the eligible articles, 1 was found to be irrelevant to the topic. Finally, 28 articles were qualified. Fig. 3 shows a workflow diagram showing the search strategy.

### Glc-induced BSA glycation

#### Oxidation products

Protein oxidation is a process of structural modification under the influence of reactive oxygen species (ROS) and reactive nitrogen species (RNS). One of the markers of protein oxidation are advanced oxidation protein products (AOPPs), formed mainly on proteins, especially albumin, and indicating oxidative stress associated with inflammatory processes^46^. Protein carbonyls (PCs) are carbonyl groups embedded in the side chains of amino acids as a result of direct oxidation or reaction with lipid peroxidation products – they are a universal indicator of protein damage^47^. Total thiols (TTs) reflect the level of free -SH groups in proteins; their decrease indicates depletion of antioxidant potential and increased oxidative stress^48^.

**Fig. 4:**
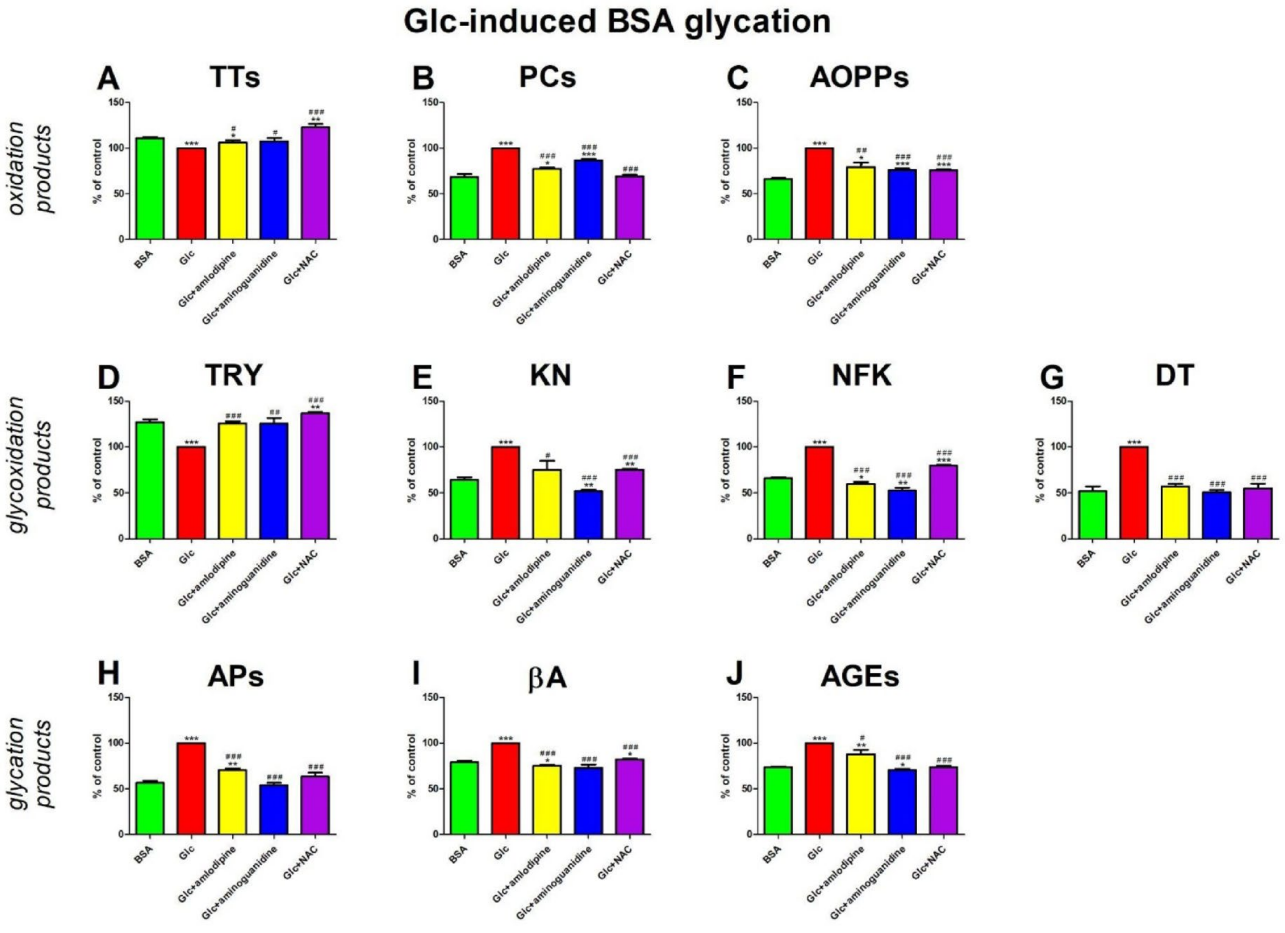
The effects of amlodipine addition to glucose (Glc)-glycated bovine serum albumin (BSA) on oxidation products (**A**–**C**), glycoxidation products (**D**–**G**), and glycation products (**H**–**J**). AGEs, advanced glycation end products; AOPPs, advanced oxidation protein products; APs, Amadori products; βA, β-amyloid; BSA, bovine serum albumin; DT, dityrosine; Glc, glucose; KN, kynurenine; NAC, N-acetylcysteine; NFK, N-formylkynurenine; PCs, protein carbonyls; Trolox, 6-hydroxy-2,5,7,8-tetramethylchroman-2-carboxylic acid; TRY, tryptophan; TTs, total thiols. *p < 0,05 vs. negative control (BSA); **p < 0,01 vs. negative control (BSA); ***p < 0,001 vs. negative control (BSA); #*p* < 0,05 vs. positive control (BSA + Glc); ##*p* < 0,01 vs. positive control (BSA + Glc); ###*p* < 0,001 vs. positive control (BSA + Glc).

The concentration of TTs was considerably higher in Glc + amlodipine (+ 6%), Glc + aminoguanidine (+ 8%), and Glc + NAC (+ 23%) than in Glc. The concentration of TTs was significantly lower in Glc (−10%) and Glc + amlodipine (−4%) versus BSA, while the level of Glc + NAC (+ 11%) was markedly higher in comparison with BSA (Fig. 4A).

The concentration of PCs was relevantly lower in Glc + amlodipine (−23%), Glc + aminoguanidine (−13%), and Glc + NAC (−31%) compared to Glc. The level of PCs was markedly elevated in Glc (+ 46%), Glc + amlodipine (+ 13%), and Glc + aminoguanidine (+ 27%) in the case of BSA (Fig. 4B).

The concentration of AOPPs was considerably decreased in Glc + amlodipine (−21%), Glc + aminoguanidine (−24%), and Glc + NAC (−24%) in relation to Glc. The level of AOPPs was effectively improved in Glc (+ 51%), Glc + amlodipine (+ 19%), Glc + aminoguanidine (+ 14%), Glc + NAC (+ 14%) in comparison to BSA (Fig. 4C).

#### Glycoxidation products

Protein glycoxidation is a process combining the mechanisms of glycation and oxidation, in which proteins are modified simultaneously by reducing sugars and ROS. This leads to the formation of damage that is more persistent and harmful than in the case of glycation or oxidation reactions alone. Tryptophan (TRY) is an amino acid particularly susceptible to oxidation and modification during glycooxidation, resulting in the loss of its fluorescent properties and changes in protein conformation^49^. Kynurenine (KN) is a product of tryptophan degradation through oxidation and photooxidation, and its increased concentration is a marker of advanced protein degradation^50^. N-formylkynurenine (NFK) is an early product of tryptophan oxidation, which often precedes the formation of kynurenine and reflects the initial stages of oxidative damage^51^. Dityrosine (DT) is formed as a result of oxidative cross-linking of tyrosine residues, which increases protein stiffness and aggregation^52^. Elevated levels of NFK, KN, and DT with reduced TRY content indicate an enhanced glycoxidation process, which may play an important role in the development of chronic, neurodegenerative diseases and diabetic complications^53^.

The content of glycoxidation products was determined using a fluorometric method. Many studies indicate that the fluorescence of glycoxidation products correlates with their concentration determined using the ELISA method^27,28,54^. Therefore, spectrofluorimetry is a fast and inexpensive alternative to immunoenzymatic tests used to assess the intensity of carbonyl stress.

The content of TRY was significantly higher in Glc + amlodipine (+ 26%), Glc + aminoguanidine (+ 26%), and Glc + NAC(+ 37%) than in Glc. The fluorescence of TRY was effectively elevated in Glc + NAC (+ 8%) compared with BSA. TRY content was significantly lower in Glc (−21%) compared to BSA (Fig. 4D).

Glc + amlodipine (−25%), Glc + aminoguanidine (−48%), and Glc + NAC (−25%) markedly reduced the KN content compared to Glc. The KN fluorescence was effectively higher in Glc (+ 56%) and Glc + NAC (+ 17%) than in BSA. Compared with BSA, KN content was efficiently inhibited in Glc + aminoguanidine (−19%) (Fig. 4E).

The content of NFK was relevantly lower in Glc + amlodipine (−40%), Glc + aminoguanidine (−47%), and Glc + NAC (−20%) than Glc. The biomarker was markedly improved in Glc (+ 52%) and Glc + NAC (+ 21%) compared to BSA. The fluorescence of NFK was significantly decreased in Glc + amlodipine (−9%) and Glc + aminoguanidine (−20%) compared to BSA (Fig. 4F).

DT content was significantly reduced in Glc + amlodipine (43%), Glc + aminoguanidine (49%), and Glc + NAC (45%) compared to BSA. The biomarker was meaningfully higher in Glc (+ 94%) than in BSA (Fig. 4G).

#### Glycation products

Protein glycation is a non-enzymatic process in which reducing sugars react with free amino groups of proteins, forming permanent structural modifications. Amadori products (APs) are early, stable products formed after the conversion of unstable Schiff bases, representing an intermediate step on the way to final protein damage^55^. β-amyloid (βA) is a peptide particularly susceptible to glycation, which promotes its aggregation and the formation of toxic deposits observed in Alzheimer’s disease^56^. AGEs are final, permanent products of glycation that can modify the physicochemical properties of proteins, increase their resistance to proteolysis, and interact with RAGE receptors, causing oxidative stress and an inflammatory response^57^. The accumulation of APs and AGEs in combination with β-amyloid glycation can lead to cellular dysfunction and the progression of neurodegenerative and metabolic diseases^58^.

The concentration of APs was markedly elevated in Glc + amlodipine (−29%), Glc + aminoguanidine (−46%), and Glc + NAC (−37%) compared to Glc. The level of APs was significantly higher in Glc (+ 77%) and Glc + amlodipine (+ 25%) than in BSA (Fig. 4H).

The content of βA was significantly decreased in Glc + amlodipine (−25%), Glc + aminoguanidine (−27%), and Glc + NAC (−18%) compared to Glc. This parameter was substantially elevated in Glc (+ 26%) and Glc + NAC (+ 4%) compared with BSA. The fluorescence of βA was effectively inhibited in Glc + amlodipine (−5%) versus BSA (Fig. 4I).

The content of AGEs was markedly lower in Glc + amlodipine (−12%), Glc + aminoguanidine (−30%), and Glc + NAC (−26%) than in Glc. The fluorescence was significantly higher in Glc (+ 35%) and Glc + amlodipine (+ 19%) than in BSA. The content of AGEs was effectively reduced in Glc + aminoguanidine (−5%) compared to BSA (Fig. 4J).

### Fru-induced BSA glycation

#### Oxidation products

**Fig. 5:**
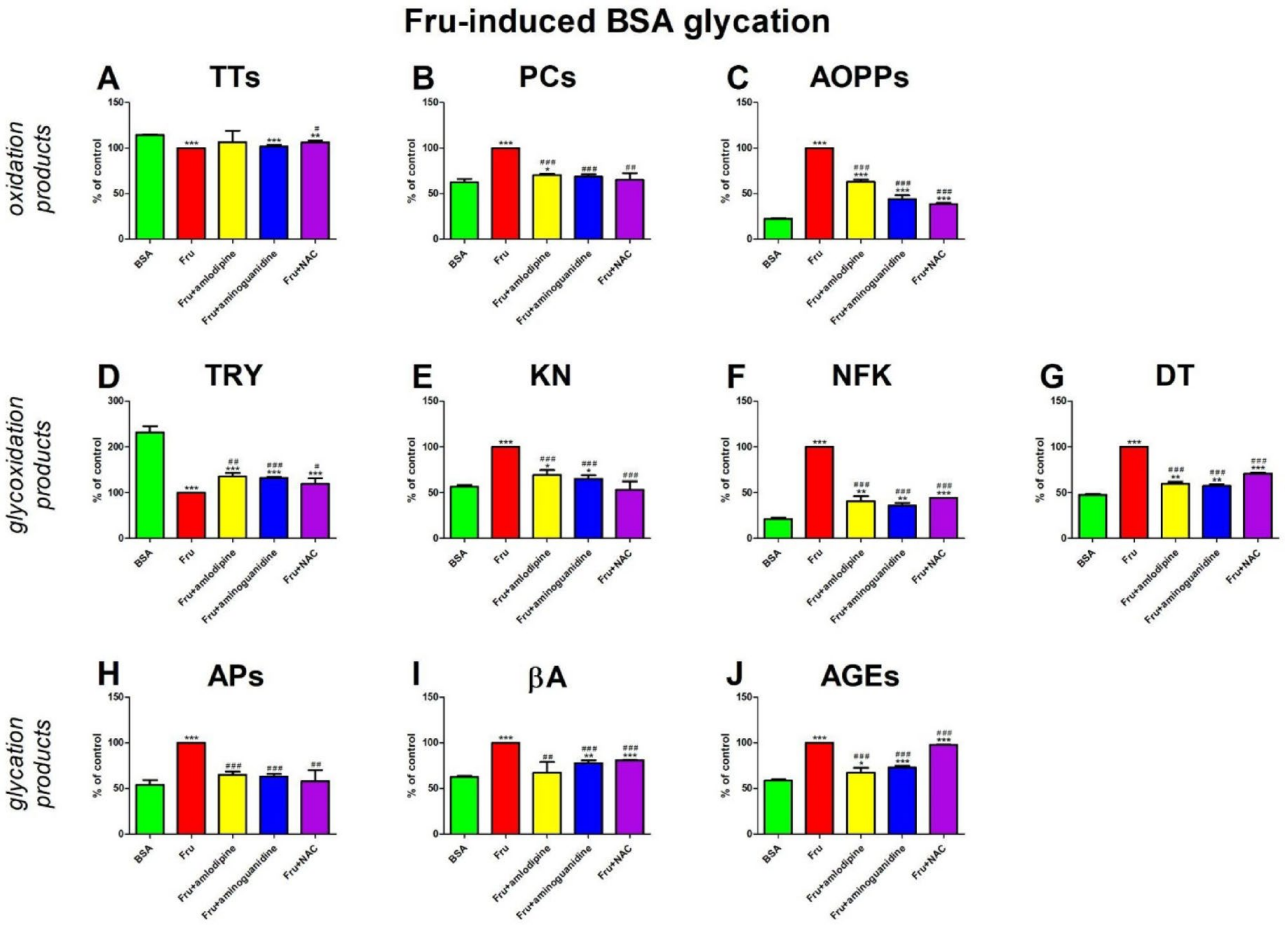
The effects of amlodipine addition to fructose (Fru)-glycated bovine serum albumin (BSA) on oxidation products (**A**–**C**), glycoxidation products (**D**–**G**), and glycation products (**H**–**J**). AGEs, advanced glycation end products; AOPPs, advanced oxidation protein products; APs, Amadori products; βA, β-amyloid; BSA, bovine serum albumin; DT, dityrosine; Fru, fructose; KN, kynurenine; NAC, N-acetylcysteine; NFK, N-formylkynurenine; PCs, protein carbonyls; Trolox, 6-hydroxy-2,5,7,8-tetramethylchroman-2-carboxylic acid; TRY, tryptophan; TTs, total thiols. *p < 0,05 vs. negative control (BSA); **p < 0,01 vs. negative control (BSA); ***p < 0,001 vs. negative control (BSA); #*p* < 0,05 vs. positive control (BSA + Fru); ##*p* < 0,01 vs. positive control (BSA + Fru); ###*p* < 0,001 vs. positive control (BSA + Fru).

The concentration of TTs was significantly higher in Fru + NAC (+ 6%) than in Fru. In comparison with BSA, the level of TTs was markedly reduced in Fru (−12%), Fru + aminoguanidine (−11%), and Fru + NAC (−7%) (Fig. 5A).

The level of PCs was relevantly inhibited in Fru + amlodipine (−30%), Fru + aminoguanidine (−32%), and Fru + NAC (−35%) versus Fru. The concentration of PCs was effectively enhanced in Fru (+ 60%) and Fru + amlodipine (+ 13%) compared to BSA (Fig. 5B).

The concentration of AOPPs was significantly lowered in Fru + amlodipine (−37%), Fru + aminoguanide (−56%), and Fru + NAC (−61%) compared to Fru. The level of AOPPs was substantially higher in Fru (+ 347%), Fru + amlodipine (+ 181%), Fru + aminoguanidine (+ 97%), and Fru + NAC (+ 72%) than in BSA (Fig. 5C).

#### Glycoxidation products

TRY content was markedly enhanced in Fru + amlodipine (+ 35%), Fru + aminoguanidine (+ 31%), and Fru + NAC (+ 19%) versus alone Fru. The fluorescence of TRY was meaningfully reduced in Fru (−57%), Fru + amlodipine (−42%), Fru + aminoguanidine (−43%), and Fru + NAC (−48%) in comparison with BSA (Fig. 5D).

The fluorescence of KN was effectively attenuated in Fru + amlodipine (−31%), Fru + aminoguanidine (−35%), and Fru + NAC (−47%) in comparison with Fru. KN content was relevantly increased in Fru (+ 77%), Fru + amlodipine (+ 22%), and Fru + aminoguanidine (+ 15%) compared to BSA (Fig. 5E).

NFK content was markedly lower in Fru + amlodipine (−59%), Fru + aminoguanidine (−64%), and Fru + NAC (−55%) than in Fru. The biomarker was significantly improved in Fru (+ 373%), Fru + amlodipine (+ 92%), Fru + aminoguanidine (+ 69%), and Fru + NAC(+ 111%) compared with BSA (Fig. 5 F).

The content of DT was substantially inhibited in Fru + amlodipine (−40%), Fru + aminoguanidine (−43%), and Fru + NAC (−29%) versus Fru. This parameter was effectively higher in Fru (+ 110%), Fru + amlodipine (+ 26%), Fru + aminoguanidine (+ 20%), and Fru + NAC (+ 48%) than BSA (Fig. 5G).

#### Glycation products

The level of APs was significantly lower in Fru + amlodipine (−35%), Fru + aminoguanidine (−37%), and Fru + NAC (−42%) compared to Fru. The concentration of APs was markedly increased in Fru (+ 86%) and Fru + amlodipine (+ 20%) in comparison with BSA (Fig. 5H).

The production of βA was effectively reduced in Fru + amlodipine (−33%), Fru + aminoguanidine (−37%), and Fru + NAC (−19%) versus Fru. The marker was relevantly higher in Fru (+ 61%), Fru + amlodipine (+ 25%), and Fru + NAC (+ 30%) compared with BSA (Fig. 5I).

The AGEs content was markedly lower in Fru + amlodipine (33%), Fru + aminoguanidine (27%), and Fru + NAC (2%) than in Fru. The Fluorescence of AGEs was significantly elevated in Fru (+ 71%), Fru + amlodipine (+ 15%), Fru + aminoguanidine (+ 24%), and Fru + NAC (+ 67%) in comparison with BSA (Fig. 5J).

### Rib-induced BSA glycation

#### Oxidation products

**Fig. 6:**
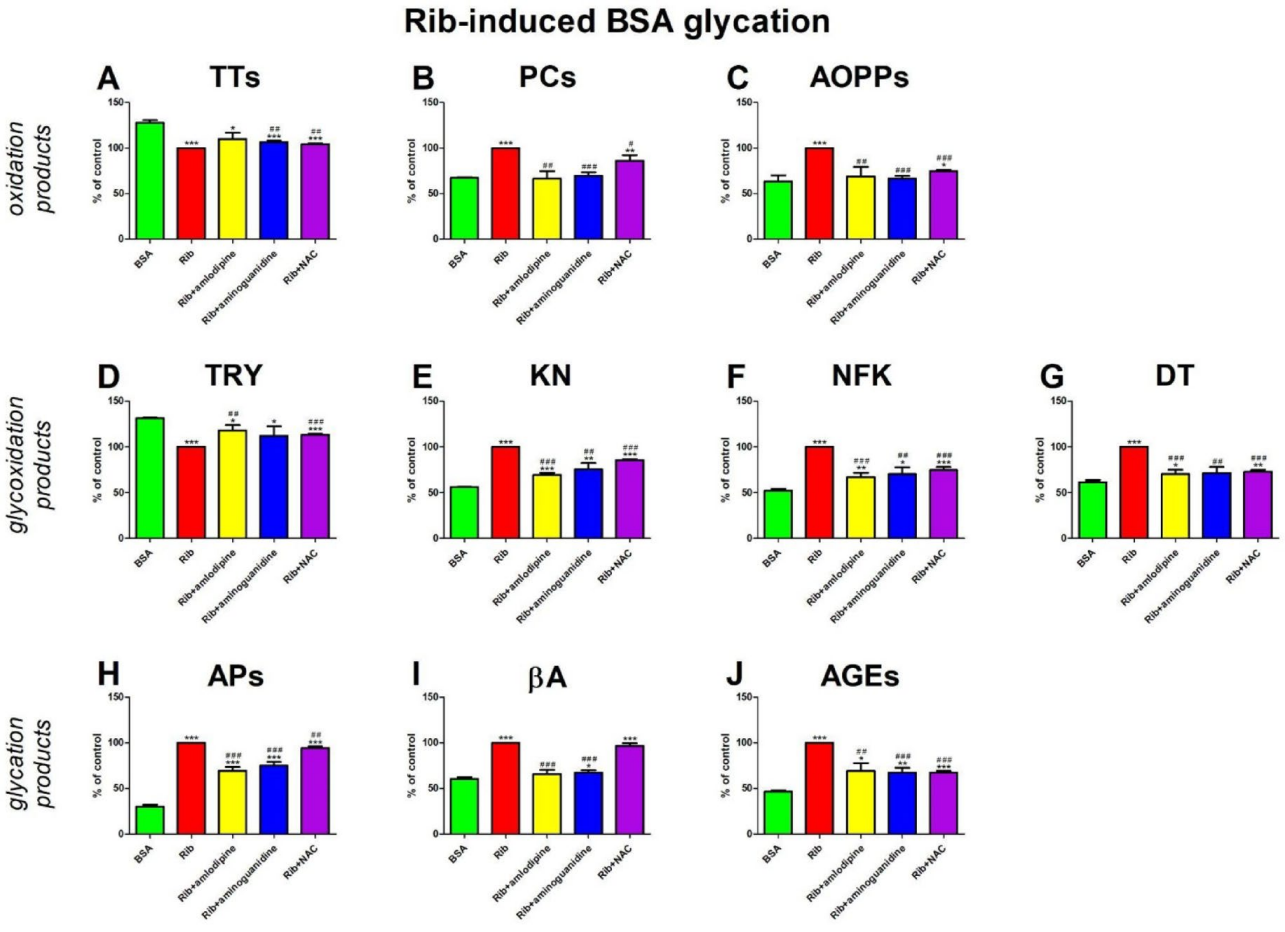
The effects of amlodipine addition to Ribose (Rib)-glycated bovine serum albumin (BSA) on oxidation products (**A**–**C**), glycoxidation products (**D**–**G**), and glycation products (**H**–**J**). AGEs, advanced glycation end products; AOPPs, advanced oxidation protein products; APs, Amadori products; βA, β-amyloid; BSA, bovine serum albumin; DT, dityrosine; KN, kynurenine; NAC, N-acetylcysteine; NFK, N-formylkynurenine; PCs, protein carbonyls; Rib, ribose; Trolox, 6-hydroxy-2,5,7,8-tetramethylchroman-2-carboxylic acid; TRY, tryptophan; TTs, total thiols. *p < 0,05 vs. negative control (BSA); **p < 0,01 vs. negative control (BSA); ***p < 0,001 vs. negative control (BSA); #*p* < 0,05 vs. positive control (BSA + Rib); ##*p* < 0,01 vs. positive control (BSA + Rib); ###*p* < 0,001 vs. positive control (BSA + Rib).

The level of TTs was relevantly enhanced in Rib + aminoguanidine (+ 6%) and Rib + NAC (+ 4%) compared to Rib alone. TTs concentration was meaningfully potentiated in Rib (−22%), Rib + amlodipine (−14%), Rib + aminoguanidine (−17%), and Rib + NAC (−18%) versus BSA (Fig. 6A).

The concentration of PCs was efficiently improved in Rib + amlodipine (−34%), Rib + aminoguanidine (−34%), and Rib + NAC (−14%) compared with Rib. The level of PCs was significantly higher in Rib (+ 49%) and Rib + NAC (+ 28%) than in BSA (Fig. 6B).

The AOPPs level was markedly elevated in Rib + amlodipine (−31%), Rib + aminoguanidine (−34%), and Rib + NAC (−25%) compared with Rib. The concentration was substantially higher in Rib (+ 58%) and Rib + NAC (+ 18%) than BSA (Fig. 6C).

#### Glycoxidation products

The content of TRY was markedly improved in Rib + amlodipine (+ 18%) and Rib + NAC (+ 13%) compared with Rib. The marker was efficiently lower in Rib (−24%), Rib + amlodipine (−10%), Rib + aminoguanidine (−15%), and Rib + NAC (−14%) than BSA (Fig. 6D).

KN content was significantly suppressed in Rib + amlodipine (−31%), Rib + aminoguanidine (−25%), and Rib + NAC (−15%) compared to Rib. The fluorescence of KN was relevantly enhanced in Rib (+ 78%), Rib + amlodipine (+ 23%), Rib + aminoguanidine (+ 34%), and Rib + NAC (+ 52%) versus BSA (Fig. 6E).

The content of NFK was effectively reduced in Rib + amlodipine (−33%), Rib + aminoguanidine (−30%), and Rib + NAC (−26%) compared with Rib. This parameter was substantially higher in Rib (+ 92%), Rib + amlodipine (+ 28%), Rib + aminoguanidine (+ 34%), and Rib + NAC (+ 42%) than in BSA (Fig. 6F).

The fluorescence of DT was effectively inhibited in Rib + amlodipine (−30%), Rib + aminoguanidine (−29%), and Rib + NAC (−27%) versus Rib. The biomarker was significantly elevated in Rib (+ 63%), Rib + amlodipine (+ 14%), and Rib + NAC (+ 18%) compared with BSA (Fig. 6G).

#### Glycation products

The level of APs was markedly lower in Rib + amlodipine (−31%), Rib + aminoguanidine (−25%), and Rib + NAC (−6%) than in Rib. APs concentration was relevantly enhanced in Rib (+ 234%), Rib + amlodipine (+ 131%), Rib + aminoguanidine (+ 150%), and Rib + NAC (+ 215%) in comparison with BSA (Fig. 6H).

βA content was meaningfully diminished in Rib + amlodipine (−34%) and Rib + aminoguanidine (−33%) compare to Rib. This parameter was significantly higher in Rib (+ 66%), Rib + aminoguanidine (+ 12%), and Rib + NAC (+ 60%) than in BSA (Fig. 6I).

The fluorescence of AGEs was substantially reduced in Rib + amlodipine (−31%), Rib + aminoguanidine (−33%), and Rib + NAC (−33%) versus Rib. AGEs content was markedly potentiated in Rib (+ 116%), Rib + amlodipine (+ 49%), Rib + aminoguanidine (+ 45%), and Rib + NAC (+ 46%) compared to BSA (Fig. 6J).

### GO-induced BSA glycation

#### Oxidation products

**Fig. 7:**
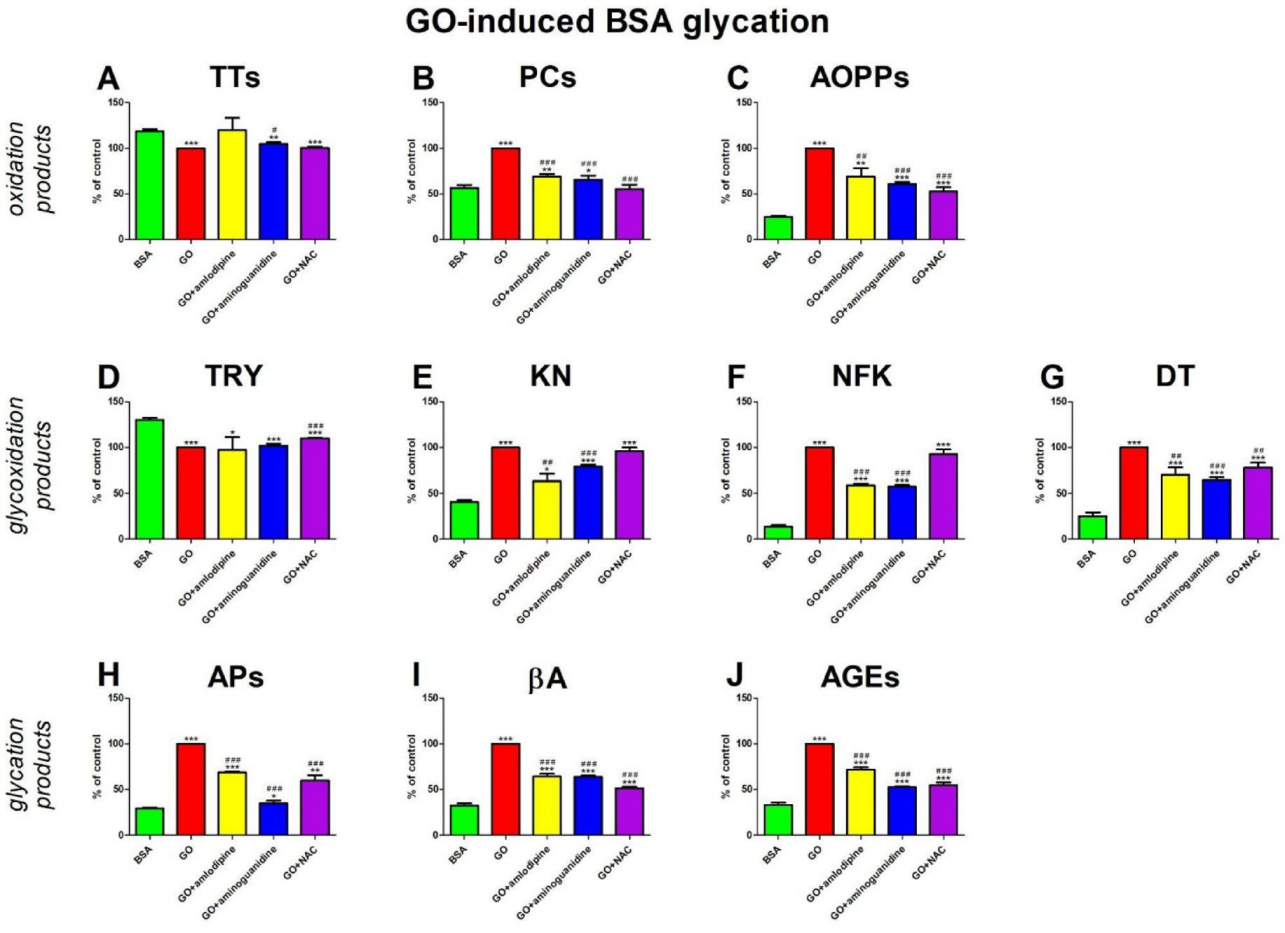
The effects of amlodipine addition to Glyoxal (GO)-glycated bovine serum albumin (BSA) on oxidation products (**A**–**C**), glycoxidation products (**D**–**G**), and glycation products (**H**–**J**). AGEs, advanced glycation end products; AOPPs, advanced oxidation protein products; APs, Amadori products; βA, β-amyloid; BSA, bovine serum albumin; DT, dityrosine; GO, glyoxal; KN, kynurenine; NAC, N-acetylcysteine; NFK, N-formylkynurenine; PCs, protein carbonyls; Trolox, 6-hydroxy-2,5,7,8-tetramethylchroman-2-carboxylic acid; TRY, tryptophan; TTs, total thiols. *p < 0,05 vs. negative control (BSA); **p < 0,01 vs. negative control (BSA); ***p < 0,001 vs. negative control (BSA); #*p* < 0,05 vs. positive control (BSA + GO); ##*p* < 0,01 vs. positive control (BSA + GO); ###*p* < 0,001 vs. positive control (BSA + GO).

The level of TTs was significantly higher in GO + aminoguanidine (+ 5%) than in GO. TTs concentration was meaningfully decreased in GO (−16%), GO + aminoguanidine (−11%), and GO + NAC (−15%) in comparison with BSA (Fig. 7A).

The concentration of PCs was considerably suppressed in GO + amlodipine (−31%), GO + aminoguanidine (−34%), and GO + NAC (−45%) versus GO. PCs level was efficiently enhanced in GO (+ 77%), GO + amlodipine (+ 22%), and GO + aminoguanidine (+ 16%) compared with BSA (Fig. 7B).

AOPPs concentration was markedly lower in GO + amlodipine (−31%), GO + aminoguanidine (−39%), and GO + NAC (−48%) than in GO alone. The marker was relevantly improved in GO (+ 302%), GO + amlodipine (+ 177%), GO + aminoguanidine (+ 145%), and GO + NAC (+ 110%) in comparison to BSA (Fig. 7C).

#### Glycoxidation products

The content of TRY was substantially higher in GO + NAC (+ 10%) than in GO. The parameter was significantly reduced in GO (−23%), GO + amlodipine (−25%), GO + aminoguanidine (−22%), and GO + NAC (−15%) compared to BSA (Fig. 7D).

KN fluorescence was significantly decreased in GO + amlodipine (−37%) and GO + aminoguanidine (−21%) compared to GO. KN content was considerably higher in GO (+ 147%), GO + amlodipine (+ 56%), GO + aminoguanidine (+ 95%), and GO + NAC (+ 136%) in comparison with BSA (Fig. 7E).

Compared to GO, the content of NFK was markedly reduced in GO + amlodipine (−41%) and GO + aminoguanidine (−43%). The biomarker was meaningfully higher in GO (+ 147%), GO + amlodipine (+ 56%), GO + aminoguanidine (+ 95%), and GO + NAC (+ 136%) than BSA (Fig. 7F).

DT content was significantly decreased in GO + amlodipine (−30%), GO + aminoguanidine (−36%), and GO + NAC (−22%) compared with GO. The fluorescence of DT was efficiently improved in GO (+ 298%), GO + amlodipine (+ 179%), GO + aminoguanidine (+ 156%), and GO + NAC (+ 210%) compared to BSA (Fig. 7G).

#### Glycation products

The APs level was effectively inhibited in GO + amlodipine (−31%), GO + aminoguanidine (−65%), and GO + NAC (−40%) compared with GO. The concentration of APs was markedly enhanced in GO (+ 244%), GO + amlodipine (+ 136%), GO + aminoguanidine (+ 20%), and GO + NAC (+ 105%) versus BSA (Fig. 7H).

The fluorescence of βA was significantly lower in GO + amlodipine (−36%), GO + aminoguanidine (−36%), and GO + NAC (−49%) than in GO. The parameter was relevantly augmented in GO (+ 209%), GO + amlodipine (+ 98%), GO + aminoguanidine (97%), and GO + NAC (+ 59%) in comparison with BSA (Fig. 7I).

The content of AGEs was markedly lower in GO + amlodipine (−28%), GO + aminoguanidine (−47%), and GO + NAC (−45%) than in GO. The marker was effectively potentiated in GO (+ 203%), GO + amlodipine (+ 117%), GO + aminoguanidine (+ 60%), and GO + NAC (+ 66%) when compared to BSA (Fig. 7J).

### ChT-induced BSA oxidation

#### Oxidation products

**Fig. 8:**
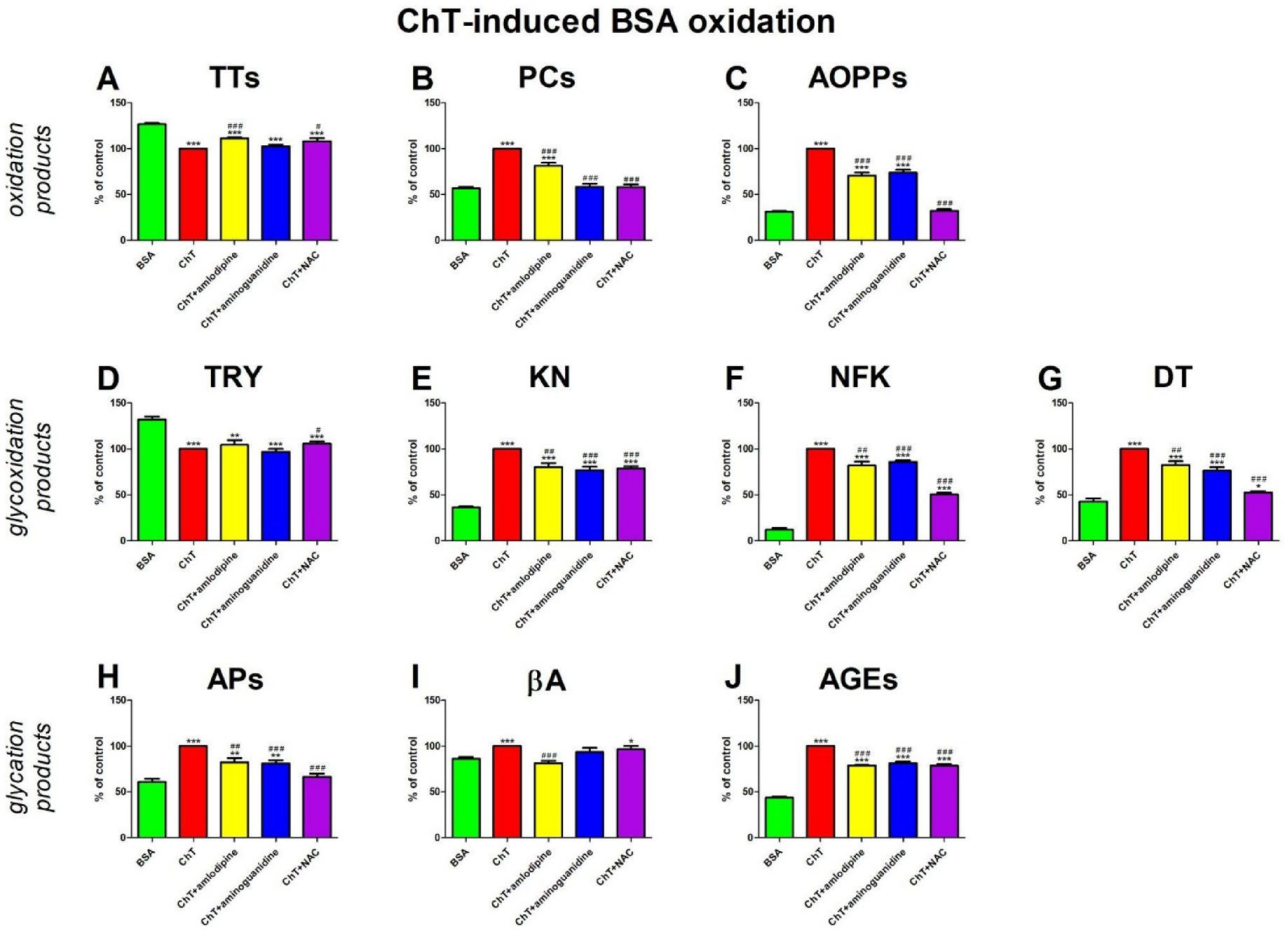
The effects of amlodipine addition to chloramine T (ChT)-glycated bovine serum albumin (BSA) on oxidation products (**A**–**C**), glycoxidation products (**D**–**G**), and glycation products (**H**–**J**). AGEs, advanced glycation end products; AOPPs, advanced oxidation protein products; APs, Amadori products; βA, β-amyloid; BSA, bovine serum albumin; ChT, chloramine T; DT, dityrosine; KN, kynurenine; NAC, N-acetylcysteine; NFK, N-formylkynurenine; PCs, protein carbonyls; Trolox, 6-hydroxy-2,5,7,8-tetramethylchroman-2-carboxylic acid; TRY, tryptophan; TTs, total thiols. *p < 0,05 vs. negative control (BSA); **p < 0,01 vs. negative control (BSA); ***p < 0,001 vs. negative control (BSA); #*p* < 0,05 vs. positive control (BSA + ChT); ##*p* < 0,01 vs. positive control (BSA + ChT); ###*p* < 0,001 vs. positive control (BSA + ChT).

The concentration of TTs was effectively higher in ChT + amlodipine (+ 11%) and ChT + NAC (+ 8%) than in ChT. In comparison with BSA, the TTs level was significantly decreased in ChT (−21%), ChT + amlodipine (−12%), ChT + aminoguanidine (−19%), and ChT + NAC (−15%) (Fig. 8A).

PCs concentration was relevantly reduced in ChT + amlodipine (−19%), ChT + aminoguanidine (−42%), and ChT + NAC (−42%) compared to ChT. The biomarker was markedly elevated in ChT (+ 76%) and ChT + amlodipine (+ 43%) versus BSA (Fig. 8B).

The level of AOPPs was significantly lower in ChT + amlodipine (−29%), ChT + aminoguanidine (−26%), and ChT + NAC (−68%) than in ChT alone. The concentration of AOPPs was substantially improved in ChT (+ 220%), ChT + amlodipine (+ 126%), and ChT + aminoguanidine (+ 137%) compared with BSA (Fig. 8C).

#### Glycoxidaton products

The content of TRY was markedly enhanced in ChT + NAC (+ 5%) compared to ChT.

The parameter was significantly inhibited in ChT (−24%), ChT + amlodipine (−21%), ChT + aminoguanidine (−27%), and ChT + NAC (−20%) compared to BSA (Fig. 8D).

KN fluorescence was relevantly attenuated in ChT + amlodipine (−20%), ChT + aminoguanidine (−23%), and ChT + NAC (−22%) versus ChT. KN content was effectively higher in ChT (+ 175%), ChT + amlodipine (+ 120%), ChT + aminoguanidine (+ 111%), and ChT + NAC (+ 116%) than BSA (Fig. 8E).

NFK content was significantly suppressed in ChT + amlodipine (−18%), ChT + aminoguanidine (−14%), and ChT + NAC (−49%) compared with ChT. This parameter was markedly potentiated in ChT (+ 733%), ChT + amlodipine (+ 580%), ChT + aminoguanidine (+ 614%), and ChT + NAC (+ 321%) compared to BSA (Fig. 8F).

DT fluorescence was relevantly lower in ChT + amlodipine (−18%), ChT + aminoguanidine (−24%), and ChT + NAC (−48%) than in ChT. The biomarker was meaningfully decreased in ChT (+ 133%), ChT + amlodipine (+ 91%), ChT + aminoguanidine (+ 77%), and ChT + NAC (+ 22%) when compared to BSA (Fig. 8G).

#### Glycation products

The APs level was effectively inhibited in ChT + amlodipine (−18%), ChT + aminoguanidine (−19%), and ChT + NAC (−34%) compared with ChT. The concentration of APs was significantly elevated in ChT (+ 64%), ChT + amlodipine (+ 35%), and ChT + aminoguanidine (+ 33%) versus BSA (Fig. 8H).

The fluorescence of βA was markedly reduced in ChT + amlodipine (−19%) compared with ChT. βA content was substantially higher in ChT (+ 16%) and ChT + NAC (+ 12%) than in BSA (Fig. 8I).

The content of AGEs was significantly attenuated in ChT + amlodipine (−21%), ChT + aminoguanidine (−19%), and ChT + NAC (−21%) compared with ChT. The marker was relevantly enhanced in ChT (+ 129%), ChT + amlodipine (+ 81%), ChT + aminoguanidine (+ 86%), and ChT + NAC (+ 80%) compared with BSA (Fig. 8J).

### Molecular docking analysis

**Fig. 9:**
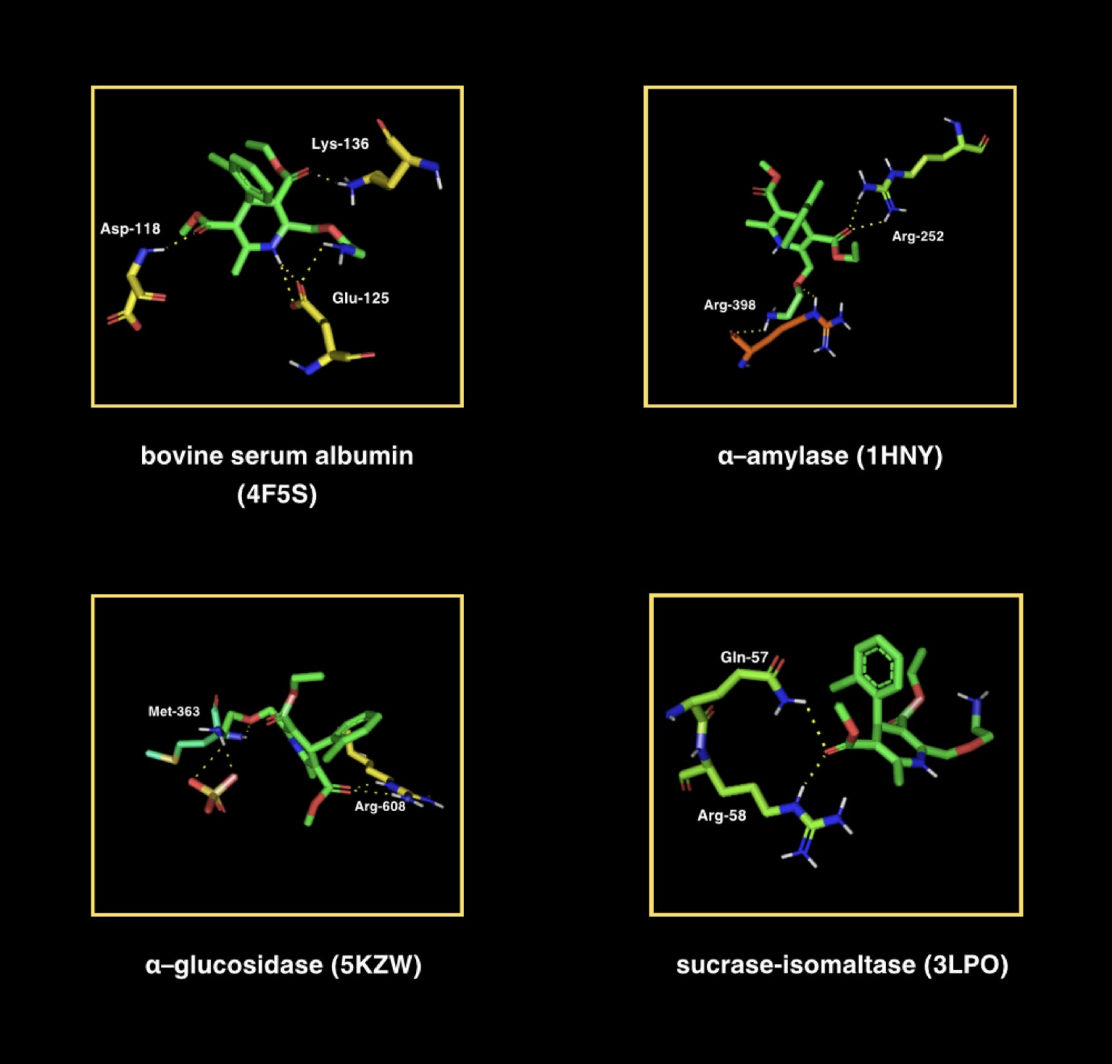
Visualization of amlodipine docking sites (modes 1) in bovine serum albumin (BSA) as well as in glycosidases: α-amylase (αA), α-glucosidase (αG), and sucrase-isolmatase (SI). The spatial structure of amlodipine has been marked in green color.

First, molecular docking simulations were conducted between amlodipine and BSA to evaluate amlodipine’s affinity for albumin binding sites (Table 1; Fig. 9). The aim was to determine if competition for these binding sites could potentially protect the protein from glycation/oxidation factors^59^. The simulation indicated that amlodipine preferentially bound to amino acids, including proline (Pro)−420, glutamic acid (Glu)−424, serine (Ser)−109, aspartic acid (Asp)−111, arginine (Arg)−458, Glu-125, alanine (Ala)−510, threonine (Thr)−514, Asp-517, Thr-518, lysine (Lys)−114, histidine (His)−145, Arg-185, leucine (Leu)−115, Glu-182, and Thr-518. In silico analysis showed that only three docking sites had root mean square deviations of atomic positions (RMSD) below 3. Amlodipine exhibited the strongest binding affinity to the BSA molecule, with a score of −5.5 kcal/mol. This site demonstrated a single polar contact involving Pro at position 420 and Glu at position 424 (Table 1; Fig. 9).

Molecular docking has also been performed between amlodipine and certain enzymes: α-amylase (αA), α-glucosidase (αG), and saccharase-isomaltase (SI). These enzymes are involved in the breakdown of polysaccharides into easily digestible simple sugars^32,60^. Amlodipine showed low binding energies to these hydrolases (−6.3, −6.2, and − 5.3 kcal/mol, respectively) (Table 2; Fig. 9). Higher affinity and thus better docking are possible when the energy of the ligand-receptor complex is lower. Amlodipine shows a strong affinity with these enzymes, which may indicate potent inhibition of their activity. Of these enzymes, αG plays the most crucial role in carbohydrate digestion. Poze 1 had the highest affinity, and also each docking site showed two polar interactions with amlodipine: (Arg-252, Arg-398), (Arg-608, methionine [Met]−363) and (Arg-58, glutamine [Gln]−57), respectively (Table 2; Fig. 9).

**Fig. 10:**
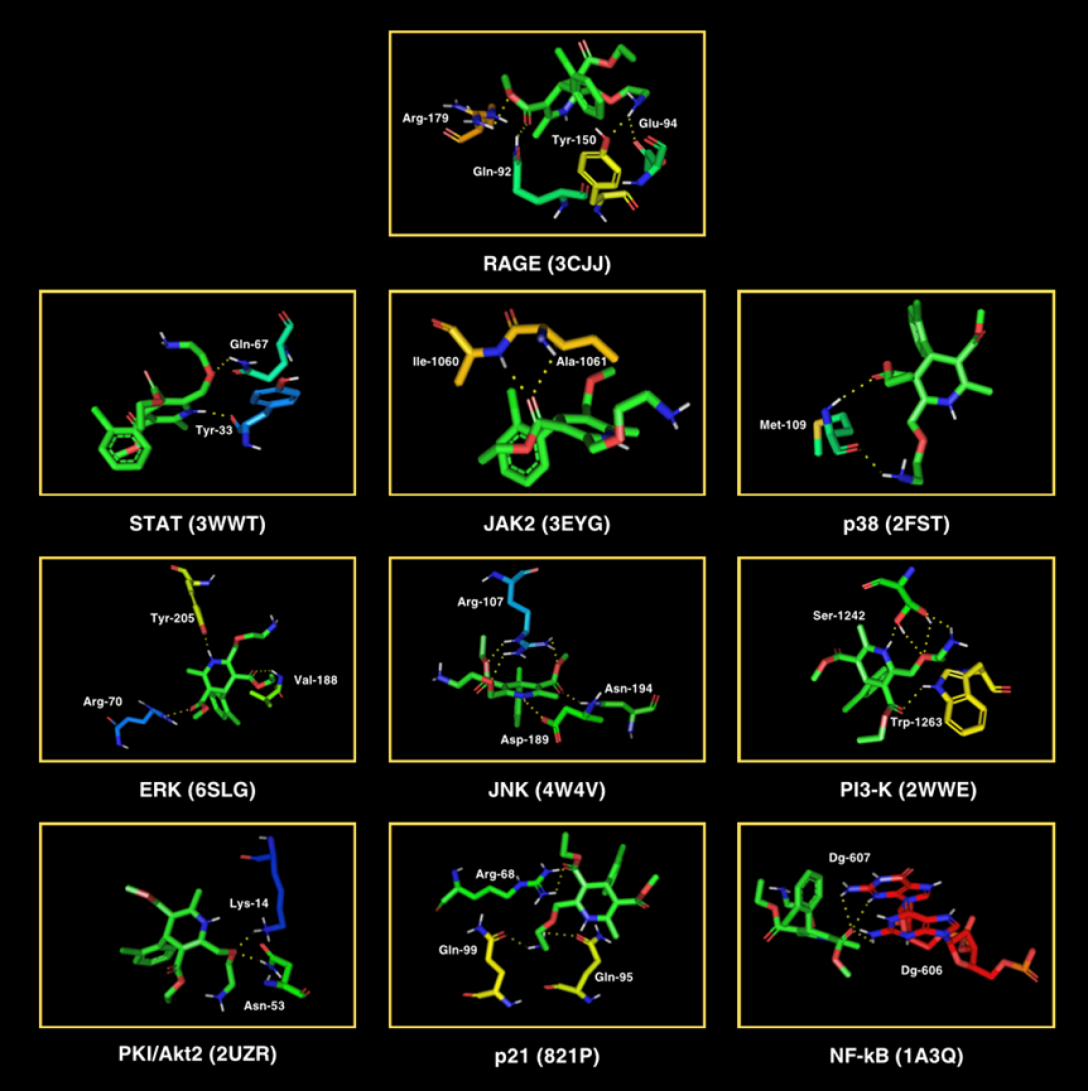
Visualization of amlodipine docking sites (modes 1) in advanced glycation end product (AGEs) pathway proteins. The spatial structure of amlodipine has been marked in green color. ERK, extracellular signal-regulated kinase; JAK2, Janus kinase 2; JNK, c-Jun N-terminal kinases; NF-κB, nuclear factor-κB; p21, protein kinase 21; p38, protein kinase 38; PI3-K, phosphatidylinositol 3-kinase; PKI/Akt2, protein kinase B/Akt serine/threonine kinase 2; rapidly accelerated fibrosarcoma; RAGE, receptor for advanced glycation end products; STAT, signal transducer and activator of transcription.

**Fig. 11:**
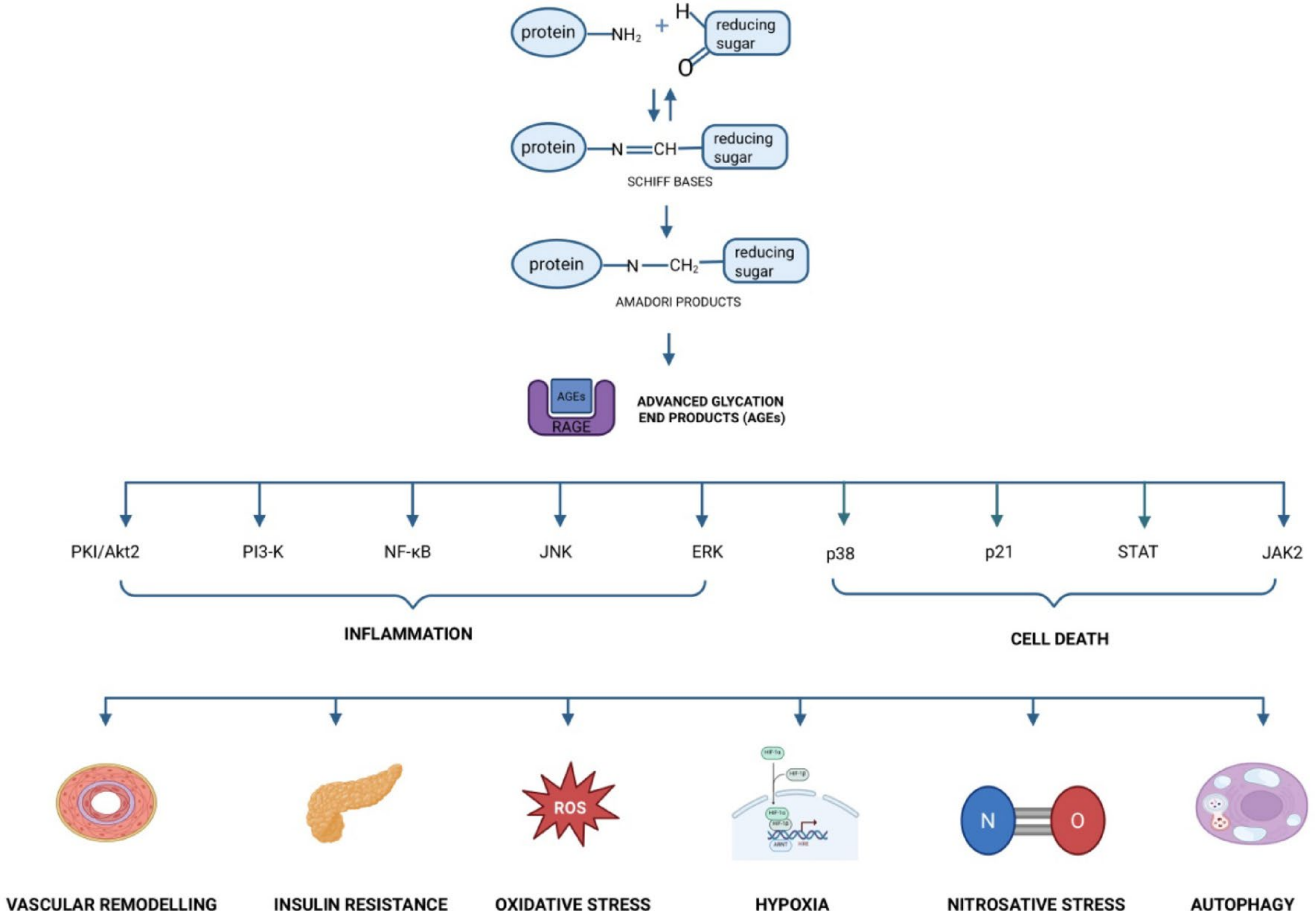
Receptor for advanced glycation end products (RAGE)/advanced glycation end product (AGE) pathway. Proteins are highlighted in green. Created with BioRender.com. AGEs, advanced glycation end products; ERK, extracellular signal-regulated kinase; JAK2, Janus kinase 2; JNK, c-Jun N-terminal kinase; NF-κB, nuclear factor-κB; p21, protein kinase 21; p38, protein kinase 38; PI3–K, phosphatidylinositol 3-kinase; PKI/Akt2, protein kinase B/Akt serine/threonine kinase 2; RAGE, receptor for advanced glycation end products; STAT, signal transducer and activator of transcription. (For interpretation of the references to color in this figure legend, the reader is referred to the Web version of this article.).

In the end, in silico docking was conducted for all proteins involved in the AGEs pathway (Table 3; Fig. 10). The AGEs/RAGE signaling pathway plays a crucial role in modulating gene transcription, which is closely Linked to the development of type 2 diabetes and its associated complications (Fig. 11). Transcription factor activity results in insulin resistance, inflammation, oxidative stress, apoptosis, and autophagy. In diabetes, overexpression of AGEs/RAGE signaling directly contributes to diabetic complications^31,61,62^. Amlodipine showed satisfactory affinity (no less than − 4.6 kcal/mol) for all target proteins. The drug strongly binds to c-Jun N-terminal kinases (JNK; −6.6 kcal/mol; three polar contacts). JNK are a family of protein kinases that play a central role in stress signaling pathways associated with gene expression, neuronal plasticity, regeneration, cell death, and regulation of cell aging. It has been shown that there is activation of the JNK pathway following exposure to various stress factors, including cytokines, growth factors, oxidative stress, and response signals to unfolded proteins or βA peptides. The primary position of amlodipine showed three polar contacts with JNK through Asn-194, Arg-107, and Asp-189 (Table 3; Fig. 10).

**Table 1:** Results of molecular docking simulations between amlodipine and bovine serum albumin (BSA). Ala, alanine; arg, arginine; asp, aspartic acid; glu, glutamic acid; his, histidine; leu, leucine; lys, lysine; pro, proline; ser, serine; thr, threonine.

Mode	Affinity (kcal/mol)	RMSD (lower bond)	RMSD (upper bond)	Amino acid residues
1	−5.5	0.000	0.000	Pro-420, Glu-424
2	−5.4	2.517	3.932	Ser-109, Asp-111, Glu-424, Arg-458
3	−5.4	31.201	33.707	Pro-420, Glu-424
4	−5.2	20.050	23.493	Glu-125
5	−5.2	22.291	24.252	Ala-510, Thr-514, Asp-517, Thr-518
6	−5.1	30.233	33.016	Ser-109, Lys-114, His-145, Arg-185, Glu-424
7	−5.0	1.572	2.224	Pro-420
8	−5.0	32.039	34.775	2x Arg-458
9	−4.9	22.621	25.450	Leu-115, Glu-182, Thr-518

**Table 2:** Results of molecular Docking simulations between amlodipine and glycosidases. Arg, arginine; gln, glutamine; met, methionine.

Enzyme number	Name of enzyme (EC number)	RCSB ID	Affinity (kcal/mol)	Number of polar contacts	Amino acid residues
1	α–amylase (αA; EC 3.2.1.1) 1HNY-7.0 1	1HNY	−6.3	2	Arg-252, Arg-398
2	α–glucosidase (αG; EC 3.2.1.20) 5KZW-6.3 4	5KZW	−6.2	2	Arg-608, Met-363
3	sucrase-isomaltase (IS; EC 3.2.1.10) 3LPO-6.0 2	3LPO	−5.3	2	Arg-58, Gln-57

**Table 3:** Results of molecular docking simulations between amlodipine and advanced glycation end products (AGEs) pathway proteins. Ala, alanine; arg, arginine; asn, asparagine; asp, aspartic acid; dG, deoxyguanosine; ERK, extracellular signal-regulated kinase; gln, glutamine; glu, glutamic acid; ile, isoleucine; JAK2, Janus kinase 2; JNK, c-Jun N-terminal kinases; lys, lysine; met, methionine; NF-κB, nuclear factor-κB; p21, protein kinase 21; p38, protein kinase 38; PI3-K, phosphatidylinositol 3-kinase; PKI/Akt2, protein kinase b/akt serine/threonine kinase 2; RAGE, receptor for advanced glycation end products; ser, serine; STAT, signal transducer and activator of transcription; trp, tryptophan; tyr, tyrosine; val, valine.

Name of protein	RCSB ID	Affinity (kcal/mol)	Number of polar contacts	Amino acid residues
Mitogen-activated protein kinases (MAPKs)				
p38	2FST	−6.2	1	Met-109
ERK	6SLG	−5.5	3	Arg-70, Tyr-205, Val-188
JNK	4W4V	−6.6	3	Asn-194, Arg-107, Asp-189
Receptors and transcription factor				
RAGE	3CJJ	−4.6	4	Arg-179, Glu-94, Gln-92, Tyr-150
STAT	3WWT	−5.0	2	Gln-67, Tyr-33
NF-kB	1A3Q	−5.9	2	dG-606, dG-607
Kinases				
PI3-K	2WWE	−5.9	2	Ser-1242, Trp-1263
PKI/Akt2	2UZR	−5.2	2	Asn-53, Lys-14
p21	821P	−4.9	3	Arg-68, Gln-95, Gln-99
JAK2	3EYG	−5.5	2	Ala-1061, Ile-1060

In order to compare the antiglycation properties of amlodipine, we also performed molecular docking for aminoguanidine and NAC (Table S2, S3, Fig. S1 in supplementary material). The docking results were much weaker than amlodipine, which may have resulted from these substances’ different mechanisms of action. Aminoguanidine neutralizes α- and β-dicarbonyl compounds that compete with glycating agents^63^. NAC binds to pro-oxidant metabolites and removes free radicals^64^. Therefore, the antiglycation effect of aminoguanidine and NAC may not be caused by such a strong interaction with AGE/RAGE proteins as with amlodipine.

## Discussion

In this study, we are the first to examine the effect of amlodipine on protein glycoxidation in vitro. For the intended purpose, we used bovine serum albumin (BSA), widely applied as a model protein to study the antiglycation properties of various substances^65,66^. BSA is frequently used in biochemical research due to its favorable price, good availability, stability, and excellent binding properties^67,68^. Both the structure of BSA and that of human albumin consist of a single amino acid chain. However, BSA has two fewer amino acids than human albumin^69^. As an essential plasma protein, albumin is vital in the human body. Among the most critical functions are transporting hormones and drugs, maintaining blood pH, and regulating colloid-oncotic pressure. Albumin can also bind transition metal ions, which gives it antioxidant properties. Under in vivo conditions, albumin is susceptible to glycoxidation due to its high plasma concentration, long half-life, and numerous amino acid residues such as lysine, arginine, and cysteine^70,71^. Since glycation in vivo takes months or years, we had to use higher concentrations of glycation factors to accelerate the process in vitro. However, it should be noted that the incubation conditions were established in kinetic studies, and the drug concentration is proportional to the high concentrations of glycation factors. Such conditions are routinely used to evaluate the antiglycation properties of new substances^27–34^.

Our research indicates that amlodipine positively impacts various aspects of protein metabolism. Specifically, it effectively prevents protein glycation, as evidenced by reduced β-amyloid (βA) structure, Amadori products (APs), and advanced glycation end products (AGEs). It also protects against albumin glycoxidation damage, as shown by elevated levels of tryptophan (TRY)and reduced fluorescence of kynurenine (KN), N-formylkynurenine (NFK), and dityrosine (DT). Proteins are especially susceptible to carbonyl stress^70,71^. Glycation occurs between the carbonyl group of reducing sugars and proteins with many free amino groups. The resulting proteins differ in both structure and biological functions. Blood glucose concentration is the factor that most intensifies protein glycation^72^. With the body’s aging or metabolic disturbances, the glycation process increases with a concomitant weakening of the elimination of glycated proteins. This situation occurs in chronic diseases such as hypertension, ischemic heart disease, and atherosclerosis^3,8,73^. Several studies have shown that accumulating AGEs and advanced oxidation protein products (AOPPs) in humans leads to tissue destruction and dysfunction^74^. These deposits can accumulate in the kidney, eye, and nervous system, as well as in the atherosclerotic plaque, exacerbating cardiovascular diseases. AGEs/AOPPs may also be responsible for micro- and macroangiopathy and progressive hypertension^75^. Indeed, binding AGEs and AOPPs to the RAGE activates genes and transcription factors that produce pro-inflammatory cytokines (TNF-α, IL-1), chemokines (CCL19, CCL-21, CXCL-8), metalloproteinases (collagenase-1 [MMP-1], gelatinase A [MMP-2]), adhesion molecules (very late antigen-1 [VLA1], CD49 antigen-like family member A/CD29 β(1)-integrin [CD49a/CD29]), cyclooxygenases 2 (COX-2), and nitric oxide synthases (NOS)^76^. Protein glycation is thus responsible for the development of inflammation, but it also disrupts extracellular matrix remodeling and intercellular signaling^77,78^. Indeed, AGEs damage collagen and elastin in the vessel walls, leading to a loss of elasticity and stiffness of the arteries^15^. The accumulation of AGEs in blood vessels also reduces NO production, resulting in increased vascular resistance and hypertension^16^. Amlodipine enhances protection against protein oxidation, as evidenced by increased total thiol (TTs) levels and decreased protein carbonyls (PCs) and AOPPs^79^. Combined with the drug, these beneficial results were consistently observed across all tested glycating and oxidizing agents (Figs. 4, 5, 6, 7 and 8). Nonetheless, certain variations in the drug’s effects can be discerned. The antiglycoxidant properties of amlodipine are more pronounced in samples containing glyoxal (GO) or chloramine T (ChT). In these combinations, the parameters exhibit more significant changes compared to the rest of the samples (e.g., the content of NFK is lower in BSA + GO/ChT + amlodipine than in BSA + Glu/Rib + amlodipine). This fact should not be surprising since different glycation agents have different kinetics of the glycation process. A very high affinity for proteins is shown by ribose, which makes it considered the strongest of the glycation agents. Therefore, both sugars (glucose [Glc], fructose [Fru], and ribose [Rib]), aldehydes (GO), and oxidants (ChT) should be used to study the antiglycation properties of various substances. The effect of amlodipine is comparable to that of standard agents with proven antiglycation (aminoguanidine) or antioxidant (N-acetylcysteine; NAC) activity. This confirms the efficacy of amlodipine in vitro. NAC is assumed to act as a disulfide bond reducer, scavenger of reactive oxygen species, and precursor of glutathione biosynthesis. In addition, NAC is converted into hydrogen sulfide and sulphane sulfur^80^. Aminoguanidine is One of the better-known compounds with antiglycation effects. Aminoguanidine reacts with dicarbonyls to form 3-amino-1,2,4-triazines. These compounds do not form cross-links and cannot further bind to proteins^81^. Furthermore, in vivo, aminoguanidine inhibits the DNA binding of NF-κB, which consequently inhibits the formation of pro-inflammatory cytokines. It has also been shown to increase anti-inflammatory mediators in several studies^82^. Unfortunately, due to its high cytotoxicity, aminoguanidine cannot be used in clinical practice^83^.

Amlodipine is a third-generation dihydropyridine (DHP) drug – an inhibitor of potential-dependent calcium channels (Fig. 1). The structural element necessary for binding to the L-type calcium channel is the NH group of the DHP ring, which forms a hydrogen bridge with glutamine (Gln-1060). The less extended ester group, whose oxygen atoms interact via hydrogen bridges with the amino acid tyrosine (Tyr-1169) and serine (Ser-1131), is also responsible for this binding^84,85^. The last One is an aromatic ring of the substituent at position 4 of DHP, which interacts with the aryl fragment of tyrosine (Tyr-1508).

This chemical structure suggests the potential antioxidant activity of amlodipine, which may explain its antiglycation properties. The literature data indicate that the amino group in the structure of amlodipine may act as a free radical acceptor and bind reactive forms of sugars, thereby inhibiting AGEs formation^86^. In addition, the dihydropyridine ring present in the amlodipine may exhibit antioxidant properties and indirectly inhibit free radical-induced glycation^87^.

Indeed, both in vitro and animal studies indicate the antioxidative action of amlodipine^88^. Pre-treatment with amlodipine, carvedilol, or dipyridamole consistently avoided cell death during hypoxia and reoxygenation^89^. Amlodipine also inhibits neuronal cell death, managing cerebrovascular stroke and neurodegenerative diseases with its antioxidant and antihypertensive activities^90^. Clinical studies also confirm the antioxidant activity of amlodipine^88^. One of these is a study conducted on patients with end-stage renal failure treated with hemodialysis, amlodipine, and valsartan. Both drug therapies significantly reduced levels of oxidized glutathione (GSSG), 8-hydroxy-2-deoxyguanosine, asymmetric dimethylarginine (ADMA), and symmetric dimethylarginine (SDMA), while amlodipine also reduced 13-hydroxy octadecadienoic acid (13-HODE) levels. It is well known that patients with end-stage renal failure suffer from increased oxidative stress, which leads to the development of cardiovascular disease. The use of amlodipine may inhibit cardiovascular complications^91^. Another clinical study compared antioxidant properties in patients with type 2 diabetes and hypertension after amlodipine and valsartan treatment. Metabolic parameters (serum glucose and insulin, lipid profile, urinary albumin, and creatinine levels) showed no significant differences before and after treatment. However, a decrease in serum nitrotyrosine levels (a marker of nitrosative protein damage) was noted, with no significant difference between amlodipine and valsartan^22^.

Molecular docking analysis was performed to judge the affinity of amlodipine for protein binding sites and to assess the ability to compete or displace other molecules (Table 1; Fig. 9). During this simulation, an affinity score between amlodipine and BSA of −5.5 kcal/mol was obtained. Molecular docking was then conducted between amlodipine and digestive enzymes whose function is to degrade polysaccharides (Table 2; Fig. 9). The enzymes used were α-amylase (αA), α-glucosidase (αG) and saccharase-isomaltase (SI), and the affinity energies were as follows: −6.3, −6.2 and − 5.3 kcal/mol. Negative binding energy values mean that the interaction between the ligand and the protein is thermodynamically favourable. The more negative the value, the stronger the affinity; in this case, amlodipine can effectively interact with carbohydrate-degrading enzymes. Therefore, the sugar-lowering property of amlodipine may be related to its antiglycation properties. Each enzyme molecule showed two polar interactions with amlodipine, and they linked through the following residues: for αA through arginine (Arg)residues: Arg-252 and Arg-398, for αG through Arg-608 and methionine (Met)−363, and for SI – Arg-58 and Gln-57. Amlodipine forms specific polar interactions (hydrogen bonds) with key amino acid residues in the active sites of enzymes. These residues are often involved in substrate binding, e.g. polysaccharides. Their occupation by amlodipine may compete with the natural substrate, consequently inhibiting enzyme activity. If amlodipine inhibits carbohydrate-digesting enzymes, it may delay the breakdown of starch and disaccharides into glucose, leading to a slower increase in postprandial glycaemia. Reduced formation of excess glucose may limit AGEs formation, confirming the anti-glycation effect of amlodipine.

The binding of AGEs to their receptor (RAGE) activates the AGEs/RAGE signaling pathway, inducing inflammation, oxidative stress, cell apoptosis, insulin resistance, and damage to pancreatic β cells^92^. Thus, AGEs/RAGE signaling is strongly associated with the evolution of type 2 diabetes and its related complications^75,93^ (Fig. 11). In the final step, molecular docking was conducted between amlodipine and AGE pathway proteins (Table 3; Fig. 10). In this docking, amlodipine demonstrated excellent binding affinity (no less than − 4.6 kcal/mol) to all ten proteins. The drug bound most strongly to N-terminal c-Jun kinases (JNK; −6.6 kcal/mol) through the following three polar contacts: asparagine (Asn)−194, Arg-107, and aspartic acid (Asp)−189. These regions are functionally important for the signalling activity of the kinase. JNK, a family of protein kinases, play a key role in stress signaling pathways^94^. They are involved in gene expression, regeneration, cell aging regulation, and cell death. Stress factors such as cytokines, growth factors, oxidative stress, and response signals to unfolded proteins and βA peptides activate the JNK pathway. It was also discovered that JNK activation is a pivotal element in regulating apoptosis signals. Amlodipine reacts with different cellular regulators, e.g., mitogen-activated protein kinases (MAPKs), transcription factors, and cell cycle proteins, which may affect the maintenance of pancreatic β-cell function and insulin sensitivity and control the cellular reply to oxidative stress and abnormal protein synthesis induced by AGEs^95^. Enhanced expression of AGEs/RAGE signaling occurs in patients with diabetes and impacts the development of diabetes-related metabolic complications (Table 3; Fig. 10).

Our systematic review indicates that the results of clinical trials remain consistent with those of animal and in vitro models. Amlodipine exhibits antioxidant properties; however, there is no data on the antiglycation activity of amlodipine. Only Nakamura et al. measured serum AGEs and soluble form of RAGE (sRAGE) in patients with chronic renal failure (stage I and II) treated with amlodipine^96^. According to their study, treatment with amlodipine did not reduce circulating AGEs and sRAGE. However, it is essential that patients with diabetes were not included in the study. Carbonyl stress has low severity in the early stages of renal failure, which may make amlodipine ineffective in lowering AGEs/RAGE. It is also possible that damage via the carbonyl stress pathway may only occur at a local level limited to renal tissue. In addition to our literature review, an animal model study has shown that amlodipine inhibits AGEs deposition in the inner membrane of the aorta, despite the absence of a “statin” effect on plasma lipids^97^. Although further research is needed, the pleiotropic properties of amlodipine may be related to its antiglycation activity. In vivo, this may be due to the fact that amlodipine is characterized by a long half-life (35–50 h) and a long duration of action^17^. However, one should not forget about the side effects of the drug, such as peripheral edema, facial flushing, pain, dizziness, palpitations, weakness, and others^98^.

### Limitations and next step

It is important to mention that our study has several limitations. Firstly, we used BSA (instead of human serum albumin) and did not examine what happens to BSA structure during the glycation process. Although the antiglycation activity of amlodipine was demonstrated in a kinetically established glycation model, only one drug concentration was tested, and the half-maximal inhibitory concentration (IC50) could not be determined.

Our in silico studies also do not provide detailed insights into the mechanisms of amlodipine action. Further experiments should evaluate structural modifications of albumin or molecular interactions of amlodipine with proteins of the AGE/RAGE pathway.

The BSA model would simplify the complicated molecular interactions between proteins in vivo, which makes it challenging to transfer the outcomes to more complicated physiological models. Indeed, in vitro studies can provide valuable preliminary information about the potential mechanisms of drug action, but they do not always translate directly into effects in living organisms. This is due to several factors that differ between the controlled conditions of in vitro studies and the complex biological processes in living organisms, including interactions between different cell types, organ systems, and environmental variables (e.g., gut microbiota or circulation) that can influence how a drug works. Amlodipine may exhibit antiglycation activity in in vitro studies, but its efficacy in living organisms may be limited by other mechanisms, such as hepatic metabolism, interactions with other drugs, or the body’s defense mechanisms. Further in vivo studies, taking into account the full biological context and environmental variables, are needed to confirm the benefits in the treatment of specific diseases^99,100^.

## Conclusions

To summarize, our study is the first to demonstrate amlodipine’s antiglycation effects. Amlodipine reduces sugar- and aldehyde-induced protein glycation to a level comparable to aminoguanidine and NAC. The in silico analysis demonstrated a strong affinity of amlodipine for αA, αG, and SI, which may explain antiglycation effects in vivo. In the docking between amlodipine and AGE pathway proteins, promising results were also obtained, and a systematic literature review confirmed the antioxidant effect of amlodipine. The antiglycation effect of amlodipine may be one of the mechanisms driving the pleiotropic effects of amlodipine, alongside its antioxidant, anti-inflammatory, and endothelial function-improving effects. Amlodipine could be used for more than just treating hypertension. Since both diabetes and hypertension involve further complications, amlodipine could potentially protect against the development of multimorbidity. In addition, the use of pleiotropic medications is associated with better adherence, as patients often have trouble following instructions and taking multiple medications^101^. Registration of a novel indication for amlodipine in the future would be a therapeutic breakthrough, particularly for people with diabetes and cardiovascular problems. Given the increased prevalence of diabetes, studies in animal models and humans are needed to confirm amlodipine’s antidiabetic mechanism of action. Due to the fact that amlodipine exhibits anti-glycation activity, its effect in diseases associated with hyperglycemia and increased oxidative stress should be investigated in both type 1 and type 2 diabetes, diabetic nephropathy, diabetic retinopathy, atherosclerosis, and hypertension^59^. Since the glycation process occurs particularly in endothelial cells, the lens, kidneys and lungs, further long-term studies should verify the effect of amlodipine on preventing organ complications.

## Supplementary Information

Below is the link to the electronic supplementary material.

Supplementary Material 1

## Data Availability

The data that support the findings of this study are available from the corresponding author upon reasonable request.
