# Supplementary material for: In vitro and in silico studies and a systematic literature review of antiglycation properties of amlodipine
Source: Sci Rep. 2025 Sep 26;15:33277. doi: 10.1038/s41598-025-18925-8 (PMC12474885; doi:10.1038/s41598-025-18925-8)
Supplement: Supplementary file 1 — Supplementary Material 1 [file 41598_2025_18925_MOESM1_ESM.docx]

**Table S1.** Multidirectional characteristics of amlodipine.

| Title | Study design | Endpoints | References |
| --- | --- | --- | --- |
| Renoprotective and antioxidant effects of cilnidipine in hypertensive patients | The study included 35 hypertensive patients, aged between 45 and 85 years, who had been on treatment with a renin-angiotensin system (RAS) inhibitor for at least three months prior to receiving either cilnidipine or amlodipine. The treatment regimens were as follows: cilnidipine was administered at an initial dose of 10 mg per day, which could be increased to a maximum daily dose of 20 mg. Amlodipine was administered at an initial dose of 5 mg per day, which could be increased to a maximum daily dose of 10 mg. No changes were made to the dosage of the RAS inhibitors during the study. All participants' target blood pressure (BP) level was 130/85 mmHg. In cases where cilnidipine or amlodipine, in combination with the RAS inhibitor, did not succeed in achieving the target BP within three months, additional antihypertensive medications, such as diuretics, were introduced to help reach the desired blood pressure level. | Following a six-month treatment with cilnidipine, there was a notable reduction in the urinary levels of 8-hydroxy-2'-deoxyguanosine (8-OHdG) when normalized to creatinine (8-OHdG/creatinine ratio), along with a significant decrease in urinary liver-type fatty-acid-binding protein (L-FABP) levels, also expressed as a ratio to creatinine (L-FABP/creatinine ratio). In contrast, these levels remained unchanged after amlodipine treatment. | ^102^ |
| Dihydropyridine calcium channel blockers inhibit non-esterified fatty-acid-induced endothelial and rheological dysfunction | In a double-masked crossover study design, eight healthy subjects were administered nifedipine, amlodipine, diltiazem, or a placebo for a period of two days prior to each study day. On the designated study days, the following assessments were conducted both before and after the infusion of lipid and heparin to elevate serum non-esterified fatty acid (NEFA) levels: endothelial function was evaluated by measuring forearm blood flow (FBF) responses to acetylcholine (ACh), leukocyte activation was assessed through ex vivo measurements of plasma myeloperoxidase (MPO) levels, adherent leukocyte numbers, and the transit time of whole blood through microchannels, oxidative stress levels were determined by analyzing plasma levels of derivatives of reactive oxygen metabolites (d-ROMs). | Following pre-treatment with a placebo, d-ROMs exhibited a time-dependent increase upon lipid/heparin infusion. However, pre-treatment with nifedipine CR substantially mitigated this increase at the 2-hour mark. Amlodipine, on the other hand, demonstrated a moderate inhibitory effect on the rise of d-ROMs. Conversely, diltiazem retard did not show a significant inhibitory effect. | ^103^ |
| Blood pressure-lowering response to amlodipine as a determinant of the antioxidative activity of small, dense HDL3 | Twenty-eight patients, comprising 17 men and 11 women, diagnosed with essential hypertension, were enrolled in the study. Each patient was administered a daily dosage of 10 mg of amlodipine, either as a standalone treatment or in combination with other antihypertensive drugs, excluding calcium antagonists. Participants were permitted to continue any previously prescribed antihypertensive, antidiabetic, or lipid-lowering medications provided they had been on a consistent regimen for a minimum of 4 weeks prior to enrollment, and this treatment remained unchanged throughout the study. The primary outcome measure focused on assessing the antioxidative activity (AOX) of high-density lipoproteins (HDL). This evaluation was based on the capacity of small, dense HDL3c particles to mitigate LDL oxidation induced in vitro by an azo initiator (AAPH). | In hypertensive patients, amlodipine treatment augmented the antioxidative capacity (AOX) of high-density lipoproteins (HDL) in individuals who experienced a blood pressure reduction surpassing the median response. This effect seems to be a consequence of the drug's hypotensive impact rather than its direct antioxidant properties. | ^104^ |
| Neurohormones and oxidative stress in nonischemic cardiomyopathy: relationship to survival and the effect of treatment with amlodipine | In the Prospective Randomized Amlodipine Survival Evaluation 2 (PRAISE-2) trial, a subgroup of 181 patients diagnosed with nonischemic cardiomyopathy were subjected to randomization into two arms: one receiving amlodipine at a dosage of 10 mg per day, and the other receiving a placebo. Blood samples were collected and assessed at three-time points: baseline, 2 weeks, and 26 weeks. The parameters examined included levels of norepinephrine, epinephrine, angiotensin II, dopamine, N-terminal pro-atrial natriuretic peptide (Nt-pro-ANP), brain natriuretic peptide (BNP), adrenolutin, and malondialdehyde. | Amlodipine does not impact circulating neurohormones and markers of oxidative stress in patients with nonischemic cardiomyopathy who are concurrently treated with angiotensin-converting enzyme inhibitors, digoxin, and diuretics. Furthermore, low levels of circulating N-terminal pro-atrial natriuretic peptide (Nt-pro-ANP) and a reduction in brain natriuretic peptide (BNP) over time indicate a favorable prognosis. | ^105^ |
| Effects of amlodipine and valsartan on oxidative stress and plasma methylarginines in end-stage renal disease patients on hemodialysis | Nineteen eligible patients, aged 18 years or above, who had been on regular thrice-weekly maintenance hemodialysis for a minimum of 3 months with a single-pool Kt/V of 41.2 and required antihypertensive therapy were included in the study. All participants successfully achieved the maximum prescribed doses of valsartan (320 mg daily) and amlodipine (10 mg daily) over 6 weeks of therapy. Among the participants, 10 received valsartan first, while nine received amlodipine initially. Data analysis was also conducted based on the study drugs' sequence, and no carry-over effects were observed.  Throughout all three phases of the study, additional antihypertensive drugs were employed to maintain the target blood pressure level. In the amlodipine and valsartan phases, drugs used during the run-in period were gradually withdrawn as needed to sustain a stable target blood pressure. The drugs utilized during the run-in, amlodipine, and valsartan phases of the study included clonidine in four, two, and two patients, labetalol in 12, 12, and 10 patients, and doxazosin in one, one, and one patient, respectively. There were no statistically significant differences between these groups. | Treatment with amlodipine and/or valsartan led to a comparable reduction in levels of asymmetric dimethylarginine (ADMA) and symmetric dimethylarginine (SDMA), regardless of blood pressure reduction. Additionally, both treatments effectively decreased, though did not completely normalize, parameters associated with oxidative stress. | ^91^ |
| Effects of valsartan or amlodipine on endothelial function and oxidative stress after one-year follow-up in patients with essential hypertension | Hypertensive patients were administered either valsartan (80–160 mg/day) or amlodipine (5–10 mg/day) for one year, with nine individuals in each treatment group. At baseline, the blood pressure levels were comparable between the two groups, and there were no significant differences in the reduction of blood pressure observed during treatment at three months, six months, or one year. Endothelial function and oxidative stress markers were assessed both before and after the treatment period. Throughout the study, patients were monitored at regular 30-day intervals. It is noteworthy that none of the patients had a diabetic condition or were active smokers. | Urinary levels of 8-isoprostane and 8-hydroxy-2'-deoxyguanosine (8-OHdG) significantly reduced in patients undergoing valsartan treatment, whereas there was no notable change in patients undergoing amlodipine treatment. The initial values of these markers were comparable between the groups treated with valsartan and amlodipine. | ^106^ |
| Olmesartan Combined With Amlodipine on Oxidative Stress Parameters in Type 2 Diabetics, Compared With Single Therapies: A Randomized, Controlled, Clinical Trial | We recruited 221 hypertensive patients with mild to moderate hypertension, type 2 diabetes mellitus, normocholesterolemic low-density lipoprotein cholesterol (LDL-C) levels below 160 mg/dL, who were overweight outpatients aged 18 or older, of either sex. These 221 patients were randomly assigned: 74 to receive olmesartan at a dosage of 20 mg/day, 72 to receive amlodipine at a dosage of 10 mg/day, and 75 to receive a fixed combination of olmesartan/amlodipine at dosages of 20/5 mg/day for 12 months. Blood pressure measurements were taken monthly, and at baseline, 6 months, and 12 months, we also assessed the following parameters: lipoprotein (a) [Lp(a)], myeloperoxidase (MPO), isoprostanes, and paraoxonase-1 (PON-1). | The fixed combination of olmesartan/amlodipine demonstrated superior effectiveness compared to individual monotherapies in reducing oxidative stress, particularly in elevating PON-1 levels and lowering Lp(a) and isoprostanes levels in patients with diabetes and hypertension. | ^24^ |
| Effect of antihypertensive treatment with candesartan or amlodipine on glutathione and its redox status, homocysteine, and vitamin concentrations in patients with essential hypertension | Forty-nine middle-aged patients, consisting of 43 men and 6 women with untreated stage I-II essential hypertension, were enrolled in a randomized, double-blind, double-dummy study. They were assigned a daily dose of either 8 mg candesartan (n = 25) or 5 mg amlodipine (n = 24) for 16 weeks. Blood pressure, reduced glutathione (GSH) and oxidized glutathione (GSSG), glutathione redox ratio (GSSG: GSH) in red blood cells, plasma homocysteine, vitamin B12, and folic acid were measured. | Untreated hypertension is associated with disrupted glutathione redox status and elevated plasma homocysteine concentrations. While both candesartan and amlodipine demonstrated beneficial effects on cellular oxidative stress, the oxidative stress status did not diminish in patients experiencing adverse changes in homocysteine levels. | ^107^ |
| Treatment of hypertension in metabolic syndrome subjects with amlodipine and olmesartan-effects on oxidized non-esterified free fatty acids and cytokine production | This study mainly recruited middle-aged hypertensive men with central obesity, borderline high fasting triglyceride levels, and low levels of HDL-C. The research followed a double-dummy parallel design employing two antihypertensive therapies to manage hypertension. The study duration was 8 weeks, with a preceding 4-week phase for diet adjustment and drug washout. A registered dietitian provided counseling on a low-sodium American Heart Association Step 2 diet, and participants were instructed to maintain this diet throughout the study. At the end of the 4-week run-in period and before randomization, subjects needed to maintain stable weight (± 5 lbs) and have systolic blood pressure values between 140–179 mm Hg or diastolic blood pressure values of 90–109 mm Hg. After a 6-week run-in period, fasting lipids and glucose were measured to determine study eligibility. Within one week, subjects returned for randomization. They were then randomly assigned to one of two treatments: olmesartan 20 mg daily or amlodipine 5 mg daily. Blood pressure was monitored every 2 weeks. If blood pressure levels remained ≥140/90 mm Hg, the amlodipine dosage was increased to 10 mg/day, and that of olmesartan was increased to 40 mg daily. For subjects with persistent high blood pressure, hydrochlorothiazide 12.5 mg was added to the treatment regimen. Fasting blood specimens were collected after a 12-hour fast upon study completion. Initially designed to randomize 50 subjects (25 olmesartan, 25 amlodipine), the trial was terminated prematurely following the withdrawal of financial support by the sponsor. This manuscript reports results on the 23 subjects who completed the trial. | Despite experimental data demonstrating that angiotensin receptor antagonists reduce cellular oxidative stress and inflammation, olmesartan did not show a significant difference from amlodipine in altering oxidized non-esterified fatty acids (ox-NEFA) and inflammatory markers in hypertensive subjects with the metabolic syndrome. | ^108^ |
| Effects of valsartan and amlodipine on oxidative stress in type 2 diabetic patients with hypertension: a randomized, multicenter study | Sixty-eight patients were enrolled in the study, comprising 48 males and 20 females. Among them, 58 subjects completed the trial. The participants were individuals aged 30 to 80 years with Type 2 diabetes and hypertension. Notably, none of the participants were currently using antihypertensive medications. They were randomly assigned to receive either amlodipine 5 mg or valsartan 80 mg every morning for 24 weeks. Throughout the 24-week treatment period, if the subjects' blood pressure did not reach the target level of 130/80 mmHg in both groups by the 16th week, an additional prescription of thiazide 12.5 mg was introduced. It is important to note that this thiazide dosage had previously been demonstrated to have no impact on oxidative stress. | Both valsartan and amlodipine demonstrated a reduction in oxidative stress markers in hypertensive patients with Type 2 diabetes. | ^22^ |
| Antioxidative effects of benidipine hydrochloride in patients with hypertension independent of antihypertensive effects. Relationship between blood pressure and oxidative stress | This study aimed to investigate the magnitude of measuring plasma levels of thiobarbituric acid reactive substances (TBARS) in patients with hypertension and compare the clinical effects of benidipine hydrochloride and amlodipine besylate on plasma TBARS. Initially, blood pressure and plasma TBARS were assessed in 85 untreated patients (48 males and 37 females, with an average age of 68 years) with at least one cardiovascular disease risk factor to examine factors influencing plasma TBARS. The results revealed that plasma TBARS were notably higher in those with hypertension, a finding that remained significant even after adjusting for other factors (r = 0.359, p < 0.01). Subsequently, among these patients, 49 individuals with hypertension or angina pectoris were administered benidipine hydrochloride at a dose of 4 mg/day. Following treatment, all patients, stratified for each factor, exhibited a significant decrease in plasma TBARS. In a parallel study, 40 untreated patients with essential hypertension were randomly allocated to either the amlodipine group (5-7.5 mg/day) or the benidipine group (4-8 mg/day) for comparison of plasma TBARS levels. | The level of plasma TBARS exhibited a marked reduction in both groups. | ^109^ |
| Therapeutic lifestyle change intervention improved metabolic syndrome criteria and is complementary to amlodipine/atorvastatin | Fifty-three volunteers diagnosed with Metabolic Syndrome (MetS) were enrolled in a randomized, placebo-controlled trial. Patients were assigned to receive either Therapeutic Lifestyle Change intervention (TLC) alone (n=26), TLC in combination with placebo, or amlodipine (5mg) /atorvastatin (10 mg) (A /A)TLC+A/A (n=27) for one year. Patients were eligible if they met the following criteria: 1) aged 40-65 years; 2) diagnosed with MetS. The levels of oxidized low-density lipoprotein cholesterol (Ox-LDL) were assessed. | Studies showed that while both TLC alone and in combination with A/A may offer benefits in improving the metabolic health of patients with Metabolic Syndrome (MetS), the addition of A/A may prevent weight gain-associated increases in Ox-LDL. Furthermore, weight loss is key in observing the TLC-associated decrease in Ox-LDL. | ^110^ |
| Improvement of endothelial function by amlodipine and vitamin C in essential hypertension | Endothelial function was assessed using venous occlusion plethysmography (VOP) in 8 hypertensive patients (age range 35 to 73 years) and 8 healthy volunteers. The hypertensive patients were treated with 5-10 mg daily amlodipine and re-evaluated for at least 2 months. Forearm blood flow (FBF) changes were measured during acetylcholine infusion through the brachial artery and with intra-arterial vitamin C. | Both acute administration of vitamin C and chronic treatment with amlodipine improved endothelial function in hypertensive patients. | ^111^ |
| Effects of olmesartan, an angiotensin II receptor blocker, and amlodipine, a calcium channel blocker, on Cardio-Ankle Vascular Index (CAVI) in type 2 diabetic patients with hypertension | Seventy patients with type 2 diabetes mellitus: 30 females and 40 males and hypertension were included in the study. Prior to this study, none of the patients had been on any antihypertensive medications. The participants were randomly assigned to two groups: one received olmesartan 10 mg/day for 12 months (n=35), and the other received amlodipine 5 mg/day for the same duration (n=35). Both medications were administered in the morning after a 12-hour fasting period. | In the current study, the angiotensin II receptor blocker (ARB) group experienced a notable reduction in 8-hydroxy-2'-deoxyguanosine (8-OHdG) levels, whereas no such decrease was noted in the calcium channel blocker group. This outcome suggests that ARB is superior to calcium channel blockers in mitigating potential oxidative stress. | ^112^ |
| Protective effects of efonidipine, a T- and L-type calcium channel blocker, on renal function and arterial stiffness in type 2 diabetic patients with hypertension and nephropathy | In this study, forty type 2 diabetic patients—14 females and 26 males—presenting with hypertension and nephropathy and receiving angiotensin receptor II blockers were recruited. They were randomly allocated into two groups: the efonidipine group (n=20) received efonidipine hydrochloride ethanolate at 40 mg/day, and the amlodipine group (n=20) received amlodipine besylate at 5 mg/day for a duration of 12 months. | The efonidipine group demonstrated noticeable reductions in plasma aldosterone levels (ALD), urinary 8-hydroxy-2'-deoxyguanosine (8-OHdG), and cardio-ankle vascular index (CAVI), whereas no such changes were demonstrated in the amlodipine group. These findings indicate that efonidipine exhibits more beneficial effects in preserving renal function, mitigating oxidative stress, and reducing arterial stiffness than amlodipine. | ^113^ |
| Olmesartan improves endothelial function in hypertensive patients: link with extracellular superoxide dismutase | This study was designed as a prospective, randomized crossover trial comprising a 4-week pre-study observation period followed by two 12-week treatment phases. Following the observation period, 31 eligible patients (including 4 women and 27 men, with a mean age of 56±11 years) were randomly allocated to either olmesartan or amlodipine treatment, with subsequent crossover to the alternate drug. For the initial 4 weeks, patients were administered 20 mg of olmesartan or 5 mg of amlodipine once daily, as per their assigned group. Patients were scheduled for visits every 4 weeks to monitor blood pressure (BP) and heart rate (HR). If BP levels did not achieve p140/90 mm Hg, the once-daily dosage was escalated to 40 mg of olmesartan or 10 mg of amlodipine. Throughout the study, other medications, including antihypertensive, antiplatelet, antidiabetic, or lipid-lowering drugs, were maintained. Baseline assessments, as well as flow-mediated vasodilation (FMD) and blood/urine sampling, were conducted prior to treatment initiation and repeated after 12 weeks of each treatment. Following this phase, treatments were swapped, and the same protocol was repeated for another 12 weeks. | Similarly, olmesartan demonstrated a reduction in serum C-reactive protein levels and an increase in urine antioxidant levels when compared to baseline measurements. Additionally, it significantly lowered urine 8-epi-prostaglandin F2α levels compared to baseline and amlodipine. While there were no significant overall alterations in plasma extracellular superoxide dismutase (EC-SOD) levels induced by either treatment, olmesartan exhibited a positive correlation between improvements in flow-mediated vasodilation (FMD) and changes in EC-SOD levels. To sum up, olmesartan effectively enhanced endothelial function in hypertensive patients, independent of its blood pressure-lowering effect. This enhancement was attributed, at least in part, to its antioxidative properties. Consequently, olmesartan may offer more significant long-term benefits for hypertensive patients with impaired endothelial function than amlodipine. | ^114^ |
| Calcium channel blocker, azelnidipine, reduces lipid hydroperoxides in patients with type 2 diabetes independent of blood pressure | The impact of azelnidipine (16mg/day) was assessed in a cohort of 14 individuals with type 2 diabetes and hypertension over a period of 12 weeks. Fasting blood samples were initially collected during the administration of amlodipine (5mg/day). Subsequently, the treatment regimen was switched from amlodipine (5mg/day) to azelnidipine (16mg/day). | Although blood pressure levels remained similar during treatment with both azelnidipine and amlodipine, azelnidipine exhibited a more significant reduction in lipid hydroperoxides within the erythrocyte membrane when compared to amlodipine. | ^115^ |
| Different effects of antihypertensive drugs on conduit artery endothelial function | The study encompassed 40 normotensive control subjects (27 male) and 180 untreated patients with essential hypertension (121 male), matched for comparison. The evaluation of endothelial function in hypertensive patients was conducted before and after a 6-month treatment period. The study involved the administration of various drugs, including two distinct calcium antagonists—nifedipine GITS (in doses ranging from 30 to 60 mg daily) and amlodipine (in doses ranging from 5 to 10 mg daily). Additionally, two different beta-blockers—atenolol (in doses ranging from 50 to 100 mg daily) and nebivolol (in doses ranging from 5 to 10 mg daily)—were employed, along with medications interfering with the renin-angiotensin system, such as the angiotensin-converting-enzyme (ACE) inhibitor perindopril (in doses ranging from 2 to 4 mg daily) and the AT1-receptor antagonist telmisartan (in doses ranging from 80 to 160 mg daily). Hydrochlorothiazide (25 mg) was added to each compound if deemed necessary. The assessment included the evaluation of brachial artery flow-mediated dilation, an endothelium-dependent response measured through high-resolution ultrasound, as well as an examination of the endothelium-independent response to glyceryl trinitrate (25 g/s). The brachial artery diameter was measured through automatic computerized analysis. A control group of forty healthy subjects was included in the study for comparison. | The administration of perindopril, telmisartan, nifedipine, and amlodipine significantly reduced oxidative stress. However, oxidative stress levels remained untouched after treatment with a blocker–based therapy. | ^116^ |
| Pleiotropic effects of cardiolipin (secondary coronary prevention) | 33 outpatients with coronary heart disease received 2,5mg, 5,0mg, and 10mg of cardiolipin daily and 75-150 mg aspirin daily for 2-month period. Nitric oxide (NO) and lipoperoxides (LPO) was measured before and after 60 days of therapy. | The effects of cardiolipin were especially expressed in restoring endothelial function and inhibiting platelet aggregation. There was a tendency to decrease the degree of hyperlipoperoxidaemia relating to oxidative stress. | ^117^ |
| Serum levels of the advanced glycation end products Nepsilon-carboxymethyllysine and pentosidine are not influenced by treatment with the angiotensin receptor II type 1 blocker irbesartan in patients with type 2 diabetic nephropathy and hypertension | This post-hoc analysis of a prospective study in patients with type 2 diabetic nephropathy aimed to investigate whether treatment with the angiotensin II type 1 receptor blocker irbesartan leads to a decrease in the serum levels of the advanced glycation end products (AGEs) pentosidine and N(epsilon)-carboxymethyllysine (CML). A total of one hundred and ninety-six patients from the Irbesartan in Diabetic Nephropathy Trial cohort (mean age 61 +/- 6.5 years, comprising 62 females and 134 males) with a mean estimated glomerular filtration rate of 47.7 ml/min underwent treatment with irbesartan (n = 65), the calcium channel blocker amlodipine (n = 61), or received a placebo (n = 70). Serum levels of pentosidine and CML were measured at baseline and after a follow-up period of 23.4 months. | The estimated glomerular filtration rate was inhibited in all groups by a mean of 8.6 ml/min. Serum levels of advanced glycation end products (AGEs) improved significantly (p < 0.001) during the follow-up period. After adjusting for renal function and total protein concentration, the changes were 53%, 55%, and 50% for pentosidine and 29%, 24%, and 23% for N(epsilon)-carboxymethyllysine (CML) in the irbesartan amlodipine, and placebo groups, respectively. The increase was not markedly dissimilar between the treatment groups. | ^118^ |
| Calcium channel blocker inhibition of AGE and RAGE axis limits renal injury in nondiabetic patients with stage I or II chronic kidney disease | In this study, we enrolled thirty non-diabetic individuals with stage I or II chronic kidney disease (CKD) and hypertension, consisting of 18 men and 12 women, with a mean age of 45.4 years. These participants were randomly divided into two groups and followed for a period of 6 months. One group received a daily dose of 16 mg azelnidipine (comprising 9 males and 6 females; IgA nephropathy n = 10, non-IgA proliferative glomerulonephritis n = 2, membranous nephropathy n = 1, unknown etiology n = 2, with a mean age of 45.3 years). The other group was administered 5 mg of amlodipine once daily (including 9 males and 6 females; IgA nephropathy n = 10, non-IgA proliferative glomerulonephritis n = 3, membranous nephropathy n = 1, unknown etiology n = 1, with a mean age of 45.5 years). | Treatment with azelnidipine, as opposed to amlodipine, reduced circulating advanced glycation end products (AGE) and their receptor (sRAGE), as well as proteinuria and urinary levels of liver-type fatty acid binding protein (L-FABP) and 8-hydroxydeoxyguanosine (8-OHdG), independent of blood pressure (BP) lowering. | ^96^ |
| Azelnidipine reduces urinary protein excretion and urinary liver-type fatty acid binding protein in patients with hypertensive chronic kidney disease | We enrolled 30 hypertensive patients with chronic kidney disease (CKD) who did not have diabetes (16 males and 14 females, average age of 47 +/- 15 years, systolic blood pressure of 156 +/- 14 mm Hg, diastolic blood pressure of 96 +/- 6 mm Hg). Individuals with a serum creatinine concentration of less than 1.5 mg/dL were selected to assess the impact on mild CKD. These patients were randomly divided into two groups and received treatment with either azelnidipine (16 mg once daily) or amlodipine (5 mg once daily). The treatment regimen was maintained for 6 months, during which clinical parameters were monitored every month, with no alteration in medication throughout the experimental period. | Azelnidipine exhibits a potent antioxidant effect, a quality not shared by amlodipine. This characteristic, coupled with its enduring antihypertensive action and low propensity for causing tachycardia, may offer an additional and noteworthy clinical advantage. | ^119^ |
| Azelnidipine and amlodipine anti-coronary atherosclerosis trial in hypertensive patients undergoing coronary intervention by serial volumetric intravascular ultrasound analysis in Juntendo University (ALPS-J) | The ALPS-J study was a prospective, randomized, open-label, blinded endpoint parallel-group investigation. One hundred and fifteen patients, comprising both males and females (with an average age of 66.2 +/- 8.7 years), diagnosed with hypertension and scheduled for elective percutaneous coronary intervention (PCI), were included in the study. Following PCI, subjects received either azelnidipine (16mg) or amlodipine (5mg). Intravascular ultrasound (IVUS) was employed to measure coronary plaque volume (PV) in non-PCI sites of the culprit vessel immediately after PCI and at the 48-week mark post-PCI. | The mean LDL-C levels at both baseline and follow-up were alike between the two groups. Azelnidipine elicited considerable decreases in high-sensitivity C-reactive protein (hs-CRP) and 8-isoprostane at follow-up compared to baseline levels, and there was a tendency towards an improvement in adiponectin. While hs-CRP and 8-isoprostane levels also showed reductions in the amlodipine group, these changes were not statistically significant. Azelnidipine demonstrated non-inferiority to amlodipine. During azelnidipine treatment, vessel volume remained unchanged, whereas lumen volume was effectively enhanced. In contrast, vessel volume and lumen volume at follow-up lowered compared to baseline in the amlodipine group. | ^120^ |
| Amlodipine: Can act as an antioxidant in patients with transfusion-dependent β-thalassemia? A double-masked, controlled, crossover trial | This study aimed to evaluate the antioxidant impacts of amlodipine in transfusion-dependent β-thalassemia (TDT) patients. This crossover trial comprised two sequences (AP and PA). In the AP sequence, nine cases got a daily dose of 5 mg amlodipine (phase I), followed by a switch to placebo (phase II). In the PA sequence, 10 patients were administered a placebo (phase I) and transitioned to amlodipine (phase II). The washout period lasted 2 weeks, and each phase was 6 months. Serum levels of malondialdehyde (MDA, μmol/L), protein carbonyl (protein CO, μM/L), glutathione (GSH, nM/L), and total antioxidant capacity (TAC, μmol FeSO4/L) were assessed at the commencement and conclusion of phases I and II. A minimum change of 5% for each outcome between amlodipine and placebo was considered clinically noticeable. | From a clinical perspective, amlodipine therapy is an effective supplementary treatment alongside conventional iron chelators, leading to improvements in MDA and TAC levels in patients with TDT. | ^86^ |
| Spironolactone and chlorthalidone in uncontrolled elderly hypertensive patients treated with calcium antagonists and angiotensin II receptor-blocker: effects on endothelial function, inflammation, and oxidative stress | Fourteen elderly Japanese patients (3 male and 11 female, age 77 ± 6 years, range 67 to 85 years) with poorly controlled essential hypertension were included in the study. They were being treated with amlodipine (5 mg/day) and candesartan (8 mg/day). They had clinic blood pressure readings (the average of three sphygmomanometric measurements) showing systolic blood pressure (SBP) >140 mmHg and/or diastolic blood pressure (DBP) >90 mmHg. Secondary hypertension was ruled out through standard clinical and laboratory tests. None of the patients had cardiac or cerebral ischemic disease, impaired renal function, diabetes mellitus, or severe hypercholesterolemia (low-density lipoprotein (LDL) cholesterol > 140 mg/dL). This was an open-label randomized crossover study comparing 16 weeks of treatment with 25 mg/day of spironolactone, an aldosterone antagonist, against 25 mg/day of chlorthalidone, a thiazide-type diuretic, both added to amlodipine and candesartan, with a 4-week washout period between treatments. | The marker of oxidative stress was oxidized LDL/b2-glycoprotein I complex (oxLDL/b2GPI), which was measured using a highly sensitive, in-house enzyme-linked immunosorbent assay system. No differences were found in the levels of LDL cholesterol, high-density lipoprotein (HDL) cholesterol, lipid peroxide, and oxLDL/b2GPI complex among the baseline condition and patients treated to chlorthalidone or spironolactone. Chlorthalidone significantly increased triglyceride levels compared to spironolactone (P < 0.05). | ^121^ |
| Losartan reduces insulin resistance by inhibiting oxidative stress and enhancing insulin signaling transduction | A total of 130 patients, both male and female, diagnosed with type 2 diabetic kidney disease (DKD), were randomly allocated to two groups. The first group received losartan (n=65) at a daily oral dose of 100 mg for a period of 12 months, while the second group received amlodipine (n=65) at a daily oral dose of 10 mg for the same duration. | The findings of this study demonstrated a significant reduction in serum urine concentrations of 8-hydroxy-2'-deoxyguanosine (8-OHdG) and nitrotyrosine (NT) levels following 12 months of losartan treatment. Additionally, the serum activity of superoxide dismutase (SOD), an antioxidative molecule, exhibited an increase with losartan treatment but not with amlodipine. | ^122^ |
| Aldosterone blockade attenuates urinary monocyte chemoattractant protein-1 and oxidative stress in patients with type 2 diabetes complicated by diabetic nephropathy | 40 outpatients with type 2 diabetes and diabetic nephropathy (23 receiving spironolactone, 14 receiving amlodipine) and 25 healthy subjects were recruited for the study. The patients were then randomly allocated to the spironolactone group (50 mg/d) or the amlodipine group (2.5 mg/d). | Amlodipine notably reduces blood pressure but does not yield a practical impact on urinary 8-Isoprostaglandin F2 alpha (8-iso-PGF2), urinary monocyte chemoattractant protein (MCP-1), or urinary albumin excretion (UAE). | ^123^ |
| Losartan vs. amlodipine treatment in elderly oncologic hypertensive patients: a randomized clinical trial | In elderly neoplastic patients, hypertension and hyperuricemia are common, both before and after undergoing chemotherapeutic treatments. This study aimed to assess the effectiveness of losartan, an antihypertensive drug known for its uricosuric properties, compared to amlodipine in hypertensive elderly patients with cancer. The trial was open-label, randomized, and comparative, spanning 30 days. A total of seventy cancer patients were randomly allocated to receive either losartan or amlodipine. | The results of this study affirm the efficacy of losartan, in contrast to amlodipine, in managing hypertension and hyperuricemia among elderly patients undergoing chemotherapy. | ^124^ |

| **Name of protein** | **RCSB ID** | **Affinity (kcal/mol)** | **Number of polar contacts** | **Amino acid residues** |
| --- | --- | --- | --- | --- |
| RAGE | 3CJJ | -3.9 | 2 | Ser-207, Thr-205 |
| JAK2 | 3EYG | -3.1 | 1 | Lys-974 |
| p38 | 2FST | -3.8 | 4 | Ser-56, Arg-57, Pro-58, Thr-68 |
| ERK | 6SLG | -2.7 | 3 | Phe-183, Thr-185, Thr-181 |
| PI3-K | 2WWE | -4.2 | 1 | Glu-1205 |
| PKI/Akt2 | 2UZR | -2.5 | 1 | Pro-42 |
| p21 | 821P | -2.6 | 2 | Cys-118, Leu-120 |
| NF-kB | 1A3Q | -3.8 | 3 | Asp-275, Glu-245, Val-288 |
| α–amylase | 1HNY | -2.5 | 2 | Asn-152, Asp-153 |
| α–glucosidase | 5KZW | -1.2 | 1 | Asp-83 |

**Table S2.** Results of molecular docking simulations between aminoguanidine and advanced glycation end products (AGEs) pathway proteins and between aminoguanidine and glycosidases. Arg, arginine; Asn, asparagine; Asp, aspartic acid; Cys, Cysteine; ERK, extracellular signal-regulated kinase; Glu, glutamic acid; JAK2, Janus kinase 2; Leu, Leucin; Lys, lysine; NF-κB, nuclear factor-κB; p21, protein kinase 21; p38, protein kinase 38; Phe, Phenylalanine; PI3-K, phosphatidylinositol 3-kinase; PKI/Akt2, protein kinase B/Akt serine/threonine kinase 2; Pro, Proline, RAGE, receptor for advanced glycation end products; Ser, serine; Thr, Threonine Val, valine.

| **Name of protein** | **RCSB ID** | **Affinity (kcal/mol)** | **Number of polar contacts** | **Amino acid residues** |
| --- | --- | --- | --- | --- |
| RAGE | 3CJJ | -3.6 | 3 | Thr-183, Gln-92, Ile-91 |
| JAK2 | 3EYG | -3.4 | 4 | Asn-971, Lys-974, Ser-961, Glu-1014 |
| p38 | 2FST | -3.6 | 1 | Glu-98 |
| ERK | 6SLG | -2.9 | 2 | Phe-183, Gly-182 |
| PI3-K | 2WWE | -4.7 | 4 | Ser-1203, Glu-1205, Asn-1295, Gln-2 |
| PKI/Akt2 | 2UZR | -2.4 | 3 | Arg-41, Glu-9, Pro-42 |
| p21 | 821P | -2.8 | 2 | Glu-126, Ser-127 |
| NF-kB | 1A3Q | -3.9 | 2 | Asp-280, Arg-290 |
| α–amylase | 1HNY | -2.3 | 1 | Ala-154 |

**Table S3.** Results of molecular docking simulations between N-acetylcysteine (NAC) and advanced glycation end products (AGEs) pathway proteins and between NAC and glycosidases. Ala, Alanine, Arg, arginine; Asn, asparagine; Asp, aspartic acid; ERK, extracellular signal-regulated kinase; Gln, Glu, glutamic acid; Gly, Glycine; Ile, Isoleucine, JAK2, Janus kinase 2; Lys, lysine; NF-κB, nuclear factor-κB; p21, protein kinase 21; p38, protein kinase 38; Phe, Phenylalanine; PI3-K, phosphatidylinositol 3-kinase; PKI/Akt2, protein kinase B/Akt serine/threonine kinase 2; Pro, Proline, RAGE, receptor for advanced glycation end products; Ser, serine; Thr, Threonine Val, valine.


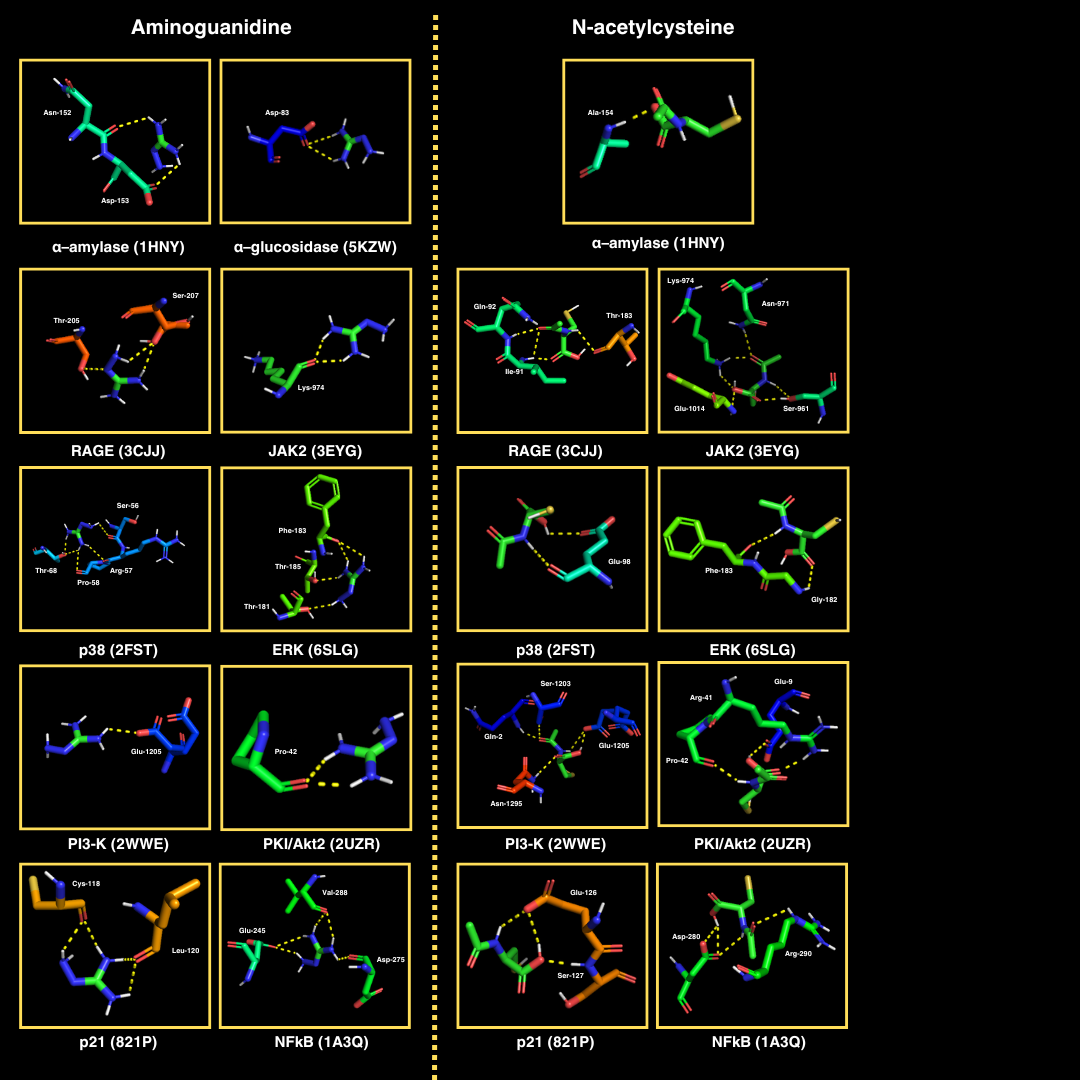
**Figure S1. V**isualization of aminoguanidine and N-acetylcysteine (NAC) docking sites (modes 1) in advanced glycation end product (AGEs) pathway proteins. Visualization of aminoguanidine and NAC docking sites (modes 1) in bovine serum albumin (BSA) as well as in glycosidases: α-amylase (αA), α-glucosidase (αG). The spatial structure of aminoguanidine and NAC have been marked in green color. Ala, Alanine; Arg, arginine; Asn, asparagine; Asp, aspartic acid; Cys, Cysteine; ERK, extracellular signal-regulated kinase; Gln, Glu, glutamic acid; Gly, Glycine; Ile, Isoleucine, JAK2, Janus kinase 2; Leu, Leucin; Lys, lysine; NF-κB, nuclear factor-κB; p21, protein kinase 21; p38, protein kinase 38; Phe, Phenylalanine; PI3-K, phosphatidylinositol 3-kinase; PKI/Akt2, protein kinase B/Akt serine/threonine kinase 2; Pro, Proline, RAGE, receptor for advanced glycation end products; Ser, serine; Thr, Threonine Val, valine.

**Reference**

101. Guiselin, T., Lecoutey, C., Rochais, C. & Dallemagne, P. Conceptual Framework of the Design of Pleiotropic Drugs against Alzheimer’s Disease. *Pharmaceutics* **15**, (2023).

102. Soeki, T. *et al.* Renoprotective and antioxidant effects of cilnidipine in hypertensive patients. *Hypertens. Res.* **35**, 1058–62 (2012).

103. Yasu, T. *et al.* Dihydropyridine calcium channel blockers inhibit non-esterified-fatty-acid-induced endothelial and rheological dysfunction. *Clin. Sci. (Lond).* **125**, 247–55 (2013).

104. Hansel, B. *et al.* Blood pressure-lowering response to amlodipine as a determinant of the antioxidative activity of small, dense HDL3. *Am. J. Cardiovasc. Drugs* **11**, 317–25 (2011).

105. Wijeysundera, H. C. *et al.* Neurohormones and oxidative stress in nonischemic cardiomyopathy: relationship to survival and the effect of treatment with amlodipine. *Am. Heart J.* **146**, 291–7 (2003).

106. Hirooka, Y., Kimura, Y., Sagara, Y., Ito, K. & Sunagawa, K. Effects of valsartan or amlodipine on endothelial function and oxidative stress after one year follow-up in patients with essential hypertension. *Clin. Exp. Hypertens.* **30**, 267–76 (2008).

107. Muda, P. *et al.* Effect of antihypertensive treatment with candesartan or amlodipine on glutathione and its redox status, homocysteine and vitamin concentrations in patients with essential hypertension. *J. Hypertens.* **23**, 105–112 (2005).

108. Rosenson, R. S. Treatment of hypertension in metabolic syndrome subjects with amlodipine and olmesartan-effects on oxidized non-esterified free fatty acids and cytokine production. *Cardiovasc. drugs Ther.* **23**, 289–94 (2009).

109. Suzuki, O. *et al.* Antioxidative effects of benidipine hydrochloride in patients with hypertension independent of antihypertensive effects. Relationship between blood pressure and oxidative stress. *Arzneimittelforschung.* **54**, 505–12 (2004).

110. Sallam, H. S., Tuvdendorj, D. R., Jialal, I., Chandalia, M. & Abate, N. Therapeutic lifestyle change intervention improved metabolic syndrome criteria and is complementary to amlodipine/atorvastatin. *J. Diabetes Complications* **34**, 107480 (2020).

111. On, Y. K. *et al.* Improvement of endothelial function by amlodipine and vitamin C in essential hypertension. *Korean J. Intern. Med.* **17**, 131–7 (2002).

112. Miyashita, Y. *et al.* Effects of Olmesartan, an Angiotensin II Receptor Blocker, and Amlodipine, a Calcium Channel Blocker, on Cardio-Ankle Vascular Index (CAVI) in Type 2 Diabetic Patients with Hypertension. *J. Atheroscler. Thromb.* **16**, 621–626 (2009).

113. Sasaki, H. *et al.* Protective effects of efonidipine, a T- and L-type calcium channel blocker, on renal function and arterial stiffness in type 2 diabetic patients with hypertension and nephropathy. *J. Atheroscler. Thromb.* **16**, 568–75 (2009).

114. Takiguchi, S. *et al.* Olmesartan improves endothelial function in hypertensive patients: link with extracellular superoxide dismutase. *Hypertens. Res.* **34**, 686–92 (2011).

115. Ohmura, C. *et al.* Calcium channel blocker, azelnidipine, reduces lipid hydroperoxides in patients with type 2 diabetes independent of blood pressure. *Endocr. J.* **54**, 805–11 (2007).

116. Ghiadoni, L. *et al.* Different effect of antihypertensive drugs on conduit artery endothelial function. *Hypertens. (Dallas, Tex. 1979)* **41**, 1281–6 (2003).

117. Kapanadze, S., Dolidze, N., Bakhutashvili, Z., Chapidze, L. & Shengelia, E. Pleiotropic effects of cardilopin (secondary coronary prevention). *Georgian Med. News* 46–8 (2005).

118. Busch, M. *et al.* Serum levels of the advanced glycation end products Nepsilon-carboxymethyllysine and pentosidine are not influenced by treatment with the angiotensin receptor II type 1 blocker irbesartan in patients with type 2 diabetic nephropathy and hypertension. *Nephron. Clin. Pract.* **108**, c291-7 (2008).

119. Nakamura, T. *et al.* Azelnidipine Reduces Urinary Protein Excretion and Urinary Liver-Type Fatty Acid Binding Protein in Patients with Hypertensive Chronic Kidney Disease. *Am. J. Med. Sci.* **333**, 321–326 (2007).

120. Kojima, T. *et al.* Azelnidipine and amlodipine anti-coronary atherosclerosis trial in hypertensive patients undergoing coronary intervention by serial volumetric intravascular ultrasound analysis in Juntendo University (ALPS-J). *Circ. J.* **75**, 1071–9 (2011).

121. Yamanari, H., Nakamura, K., Miura, D., Yamanari, S. & Ohe, T. Spironolactone and chlorthalidone in uncontrolled elderly hypertensive patients treated with calcium antagonists and angiotensin II receptor-blocker: effects on endothelial function, inflammation, and oxidative stress. *Clin. Exp. Hypertens.* **31**, 585–94 (2009).

122. Pan, Y. *et al.* Losartan reduces insulin resistance by inhibiting oxidative stress and enhancing insulin signaling transduction. *Exp. Clin. Endocrinol. Diabetes* **123**, 170–7 (2015).

123. Takebayashi, K., Matsumoto, S., Aso, Y. & Inukai, T. Aldosterone blockade attenuates urinary monocyte chemoattractant protein-1 and oxidative stress in patients with type 2 diabetes complicated by diabetic nephropathy. *J. Clin. Endocrinol. Metab.* **91**, 2214–7 (2006).

124. Motta, M. *et al.* Losartan vs. amlodipine treatment in elderly oncologic hypertensive patients: a randomized clinical trial. *Arch. Gerontol. Geriatr.* **53**, 60–3 (2011).
